# Urinary tract infections trigger synucleinopathy via the innate immune response

**DOI:** 10.1007/s00401-023-02562-4

**Published:** 2023-03-30

**Authors:** Wouter Peelaerts, Gabriela Mercado, Sonia George, Marie Villumsen, Alysa Kasen, Miguel Aguileta, Christian Linstow, Alexandra B. Sutter, Emily Kuhn, Lucas Stetzik, Rachel Sheridan, Liza Bergkvist, Lindsay Meyerdirk, Allison Lindqvist, Martha L. Escobar Gavis, Chris Van den Haute, Scott J. Hultgren, Veerle Baekelandt, J. Andrew Pospisilik, Tomasz Brudek, Susana Aznar, Jennifer A. Steiner, Michael X. Henderson, Lena Brundin, Magdalena I. Ivanova, Tom J. Hannan, Patrik Brundin

**Affiliations:** 1 Department of Neurodegenerative Science, Parkinson’s Disease Center, Van Andel Institute, Grand Rapids, MI USA; 2 Laboratory for Neurobiology and Gene Therapy, Department of Neurosciences, KU Leuven, Louvain, Belgium; 3 Laboratory for Virology and Gene Therapy, Department of Pharmacy and Pharmaceutical Sciences, KU Leuven, Louvain, Belgium; 4 Center for Clinical Research and Disease Prevention, Bispebjerg and Frederiksberg Hospital, Copenhagen, Denmark; 5 Department of Neurology, University of Michigan, Ann Arbor, MI USA; 6 Present Address: Neuroscience Graduate Program, Northwestern University Feinberg School of Medicine, Chicago, IL USA; 7 Flow Cytometry Core Facility, Van Andel Institute, Grand Rapids, MI USA; 8 Leuven Viral Vector Core, Department of Neurosciences, KU Leuven, Louvain, Belgium; 9 Department of Pathology and Immunology, Washington University School of Medicine, St. Louis, MO USA; 10 Department of Epigenetics, Van Andel Institute, Grand Rapids, MI USA; 11 Centre for Neuroscience and Stereology, Bispebjerg and Frederiksberg Hospital, Copenhagen, Denmark; 12 Biophysics Program, University of Michigan, Ann Arbor, MI USA; 13 Pharma Research and Early Development (pRED), F. Hoffmann-La Roche, Basel, Switzerland

## Abstract

Symptoms in the urogenital organs are common in multiple system atrophy (MSA), also in the years preceding the MSA diagnosis. It is unknown how MSA is triggered and these observations in prodromal MSA led us to hypothesize that synucleinopathy could be triggered by infection of the genitourinary tract causing ɑ-synuclein (ɑSyn) to aggregate in peripheral nerves innervating these organs. As a first proof that peripheral infections could act as a trigger in MSA, this study focused on lower urinary tract infections (UTIs), given the relevance and high frequency of UTIs in prodromal MSA, although other types of infection might also be important triggers of MSA. We performed an epidemiological nested-case control study in the Danish population showing that UTIs are associated with future diagnosis of MSA several years after infection and that it impacts risk in both men and women. Bacterial infection of the urinary bladder triggers synucleinopathy in mice and we propose a novel role of ɑSyn in the innate immune system response to bacteria. Urinary tract infection with uropathogenic *E.* *coli* results in the de novo aggregation of ɑSyn during neutrophil infiltration. During the infection, ɑSyn is released extracellularly from neutrophils as part of their extracellular traps. Injection of MSA aggregates into the urinary bladder leads to motor deficits and propagation of ɑSyn pathology to the central nervous system in mice overexpressing oligodendroglial ɑSyn. Repeated UTIs lead to progressive development of synucleinopathy with oligodendroglial involvement in vivo. Our results link bacterial infections with synucleinopathy and show that a host response to environmental triggers can result in ɑSyn pathology that bears semblance to MSA.

**Supplementary Information:**

The online version contains supplementary material available at 10.1007/s00401-023-02562-4.

## Introduction

Multiple system atrophy (MSA) is a rare and fatal neurodegenerative disease of unknown origin [15, 46]. MSA progressively affects a wide variety of motor and autonomous nervous system functions and invariably leads to severe disability and death [29]. Some of the earliest features in MSA include erectile and urinary bladder dysfunction, which can debut months to years before the onset of motor symptoms [4, 35]. It is estimated that over 20% of MSA patients experience lower urogenital symptoms as their initial complaint [56, 57], suggesting that in some cases, the disease might start within these sites [47].

The presence of aggregated ɑSyn in brain oligodendrocytes is a pathognomonic feature of MSA [21, 50]. Oligodendrocytes normally express low levels of ɑSyn [3, 13, 14, 28]. Aggregation of ɑSyn in oligodendrocytes leads to the assembly of disease-specific ɑSyn fibrils with highly aggregation-prone features [52, 62]. In MSA patients, aggregates of ɑSyn are also detected in spinal cord oligodendrocytes and Schwann cells of the spinal nerves [6, 42]. Recently, it was shown that aggregates of ɑSyn can propagate from the urinary tract to the spinal cord in ɑSyn transgenic mice [12].

We and others hypothesized that synucleinopathy can be triggered peripherally by a pathogen [27, 63]. We proposed that the trigger occurs during the disease prodrome, with the first steps being a local or systemic host immune response. The idea that ɑSyn might participate in the regulation of immune responses has been proposed recently [37], but its precise role in that context remains elusive. Urinary tract infections (UTIs) are among the most common bacterial infections in humans [18] and are prominent in MSA, both before and after formal diagnosis of MSA [48, 49]. It is estimated that more than half of MSA patients suffer from recurrent or chronic UTIs [49] and which can lead to urosepsis and death [48]. Given the relevance of UTIs in MSA, we decided to investigate if UTIs could trigger the disease. However, we want to emphasize that other infectious triggers in the urogenital or gastrointestinal system might elicit similar responses, and due to shared innervation between organs, it is conceivable that infections in different tissues could trigger synucleinopathies with similar central nervous system distributions.

Given the high frequency of UTIs in prodromal MSA, we performed a nested-case control study based on all MSA cases in the Danish population (over 5 million individuals) to investigate a potential association between UTIs and MSA. We found that UTIs are associated with a significant risk increase for a subsequent diagnosis of MSA several years after infection. Aggregated ɑSyn is present in the nerves of the urinary bladder and its vasculature of normal subjects and MSA patients. We show that in mice uropathogenic *E. coli* (UPEC) cause infiltration of neutrophils that release ɑSyn and form insoluble aggregates during UTI. Following injection of MSA-derived ɑSyn aggregates into the urinary bladder of humanized ɑSyn transgenic mice, bladder dysfunction, motor deficits with oligodendroglial ɑSyn pathology all progressively develop in the CNS resulting in an animal model which mimics features of MSA. Repeated UTIs triggered central oligodendroglial ɑSyn pathology in humanized ɑSyn transgenic mice. Thus, we here show epidemiological data linking UTIs with a later diagnosis of MSA and present a potential mechanistic pathway in which a common pathogen elicits the formation of ɑSyn aggregates in the urinary bladder, which in turn can propagate into the CNS, leading to progressive development of neuropathology.

## Results

### A population-based case–control study of UTIs and MSA

To date, no environmental risk factors of MSA have been identified. To examine if UTIs could act as a trigger of MSA in the disease prodrome, we performed a population-based case–control study testing if an association between UTIs and MSA might exist (Supplementary Table 1, online version). The cohort consisted of all Danish citizens alive in 2016, approximately 5 million individuals. We identified 227 people that were diagnosed with MSA in the period 2003–2018. To avoid survival bias, we excluded 119 diagnosed before 2016, leaving 108 cases diagnosed with MSA in the period 2016–2018 to analyze. Among the 108 identified MSA cases, 52 (48%) were female and 56 (52%) were male. The mean age at date of MSA diagnosis was 69.1 years (95% CI 68.3–70.0) for female patients and 67.3 years (95% CI 66.3–68.2) for male patients.

The prodrome in MSA is relatively short and to minimize the likelihood of including UTIs that were a consequence of impaired bladder emptying or neurogenic lower urinary tract dysfunction, we excluded UTIs that occurred in the first 2 years before diagnosis from our analysis. At 2–8 years before MSA diagnosis, 48.2% of cases and 23.4% of controls had an UTI registered (OR 3.04, 95% CI 2.03–4.54; *p* < 0.001) (Table 1). Most registrations of UTIs occurred within 2 to 4 years before the MSA diagnosis (OR 3.57; 95% CI 2.36–5.41; *p* < 0.001), although the OR of UTIs was already higher in the MSA cases compared with the control group at 5 to 8 years (OR 1.79; 95% CI 1.10–2.90; *p* = 0.02) before MSA diagnosis (Table 1). Having recurrent UTIs was more frequent in MSA cases (24.1%) than controls (7.8%), giving an OR of 3.76 (95% CI 2.29–6.16; *p* < 0.001) (Table 1). Females are more likely to have a UTI (63.9%) than males (36.1%) (*p* value of Chi-square test < 0.001). However, the odds for UTI before MSA do not differ between females and males (Supplementary Table 2, online version). Adding urosepsis as a variable to the analysis does not change these estimates significantly (Supplementary Table 3, online version). These results show that after a UTI, the odds of MSA diagnosis is around threefold greater, tentatively suggesting that UTIs are a MSA risk factor for both men and women.

**Table 1:** The impact of urinary tract infections on multiple system atrophy

Analysis	MSA cases (%)	Controls (%)	OR (95% CI)*	*p* value
2–8 years	52 (48.2%)	253 (23.4%)	3.04 (2.03–4.54)	< 0.001
2–4 years	45 (41.7%)	180 (16.7%)	3.57 (2.36–5.41)	< 0.001
5–8 years	24 (22.2%)	149 (13.8%)	1.79 (1.10–2.90)	0.02
Relapse 2–8 years	26 (24.1%)	84 (7.8%)	3.76 (2.29–6.16)	< 0.001

*Subjects having at least one UTI in the specified period before MSA diagnosis date

### Detection of pathological ɑSyn in human urinary bladder

**Fig. 1:**
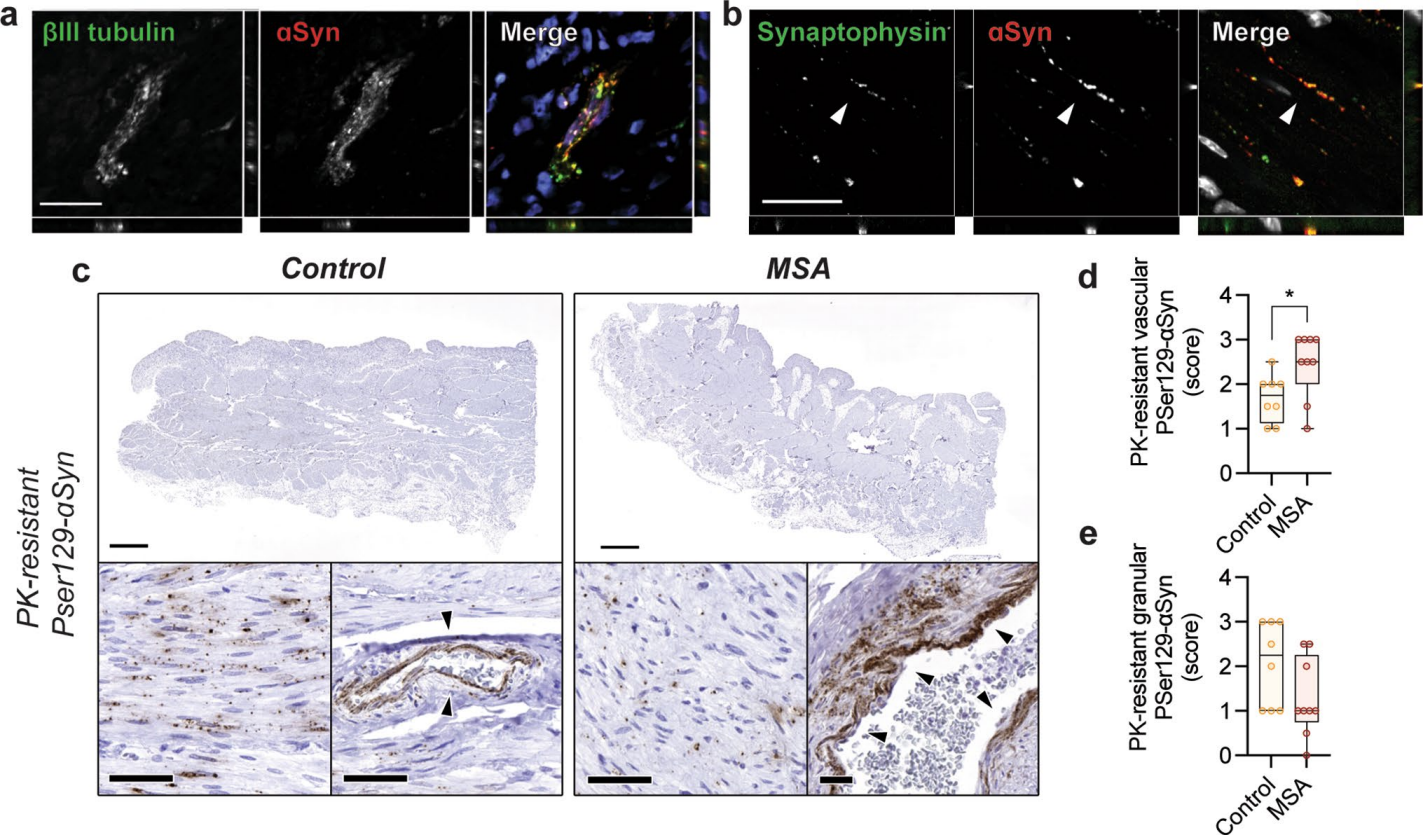
Detection of pathological ɑSyn in the urinary bladder of MSA patients and controls. Immunohistochemical analysis of endogenous ɑSyn in human urinary bladder shows ɑSyn expression in **a** βIII tubulin-positive intramural ganglia and **b** neuronal synapses (white arrows). For the detection of pathological ɑSyn urinary bladder tissue was treated with proteinase K (PK) and stained for PSer129- ɑSyn. **c** PK-resistant PSer129-ɑSyn is detected in urinary bladder tissue of both controls and MSA patients. A granular pattern of PK-resistant PSer129-ɑSyn is observed throughout the detrusor and lamina propria in addition to a vascular pattern where ɑSyn deposits around the vascular epithelium (black arrows) (scale bar represents 2 mm for overview figures and 50 µm for the detailed panels). The PSer129-⍺Syn immunostaining is localized to the perivascular area in the control subject whereas it extends more deeply into perivascular space for the MSA case. **d** Semi-quantitative analysis of PK-resistant PSer129-ɑSyn pathology in controls and cases shows significantly more vascular pathology for MSA cases (*n* = 8, *p = 0.02 with a non-parametric Mann–Whitney unpaired two-tailed *t* test) but **e** no differences in the granular PSer129-ɑSyn is detected (*n* = 8, *p* > 0.05 with a non-parametric Mann–Whitney unpaired two-tailed *t* test)

Recent studies have suggested that ɑSyn is involved in peripheral immunity [2, 60], but its precise role remains unclear. To explore where ɑSyn is expressed in the urinary bladder and whether pathological aggregated ɑSyn might be present, we performed immunohistochemistry in human urinary bladder. The urinary bladder is densely innervated by sensory and somatic fibers in the suburothelial and urothelial layers [17]. We detected widespread and punctuate endogenous ɑSyn expression within intramural ganglia (Fig. 1a) and neuronal synapses (Fig. 1b) throughout the detrusor muscle and in the lamina propria close to the bladder lumen.

Recently, it was shown that pSer129-ɑSyn-immunoreactive are present in MSA biopsied urinary bladder tissue [12]. To further expand on these findings, we examined paraffin-embedded postmortem urinary bladder tissue from 8 MSA patients and 8 age- and sex-matched controls (Table 1) using antibodies to detect total pSer129-ɑSyn and conformation-specific high molecular weight forms of ɑSyn (FILA-1). Interestingly, we found positive immunohistochemical staining in both control and MSA cases, displaying a granular pattern of staining throughout bladder within the detrusor muscle and lamina propria, suggesting that it is expressed in neurons (Supplementary Fig. 1a, online version). In addition, we found dense accumulation of both ɑSyn markers around blood vessels (Supplementary Fig. 1b, online version). By quantifying ɑSyn immunofluorescence, we determined that control and MSA subjects have no detectable differences in ɑSyn particle number or size (Supplementary Fig. 1c–e, online version).

Next, to examine if aggregated pSer129-ɑSyn is present, we pretreated paraffin-embedded urinary bladder tissue with proteinase K (PK) to remove soluble forms of pSer129-ɑSyn. Staining for insoluble, PK-resistant, Pser129-ɑSyn shows a similar general pattern of distribution as pSer129-ɑSyn staining, but it is more pronounced around perivascular regions (Fig. 1c). Using established protocols [11], we performed semi-quantitative scoring of PK-resistant Pser129-ɑSyn pathology and observed a change in distribution of insoluble Pser129-ɑSyn, with MSA cases exhibiting significantly more staining around blood vessels, possibly suggestive of a role for aggregated ɑSyn in perivascular inflammation in MSA (Fig. 1d, e).

Together, this shows that ɑSyn is expressed throughout the urinary bladder, that aggregated Pser129-ɑSyn-immunoreactive ɑSyn can be present in the urinary bladder of both control subjects and MSA patients but that the distribution of pathological ɑSyn within the bladder wall is altered in MSA. Given the association between UTI and MSA (Table 1) and the perivascular accumulation of pathological ɑSyn (Fig. 1d, e), it raises the possibility that ɑSyn aggregation of could be triggered by infections. This opens for the possibility that in the presence of disease-specific facilitators, aggregated ɑSyn is potentially set to propagate via peripheral nerves from the genitourinary tract to the CNS.

### Urinary tract infections trigger aggregation of endogenous ɑSyn

**Fig. 2:**
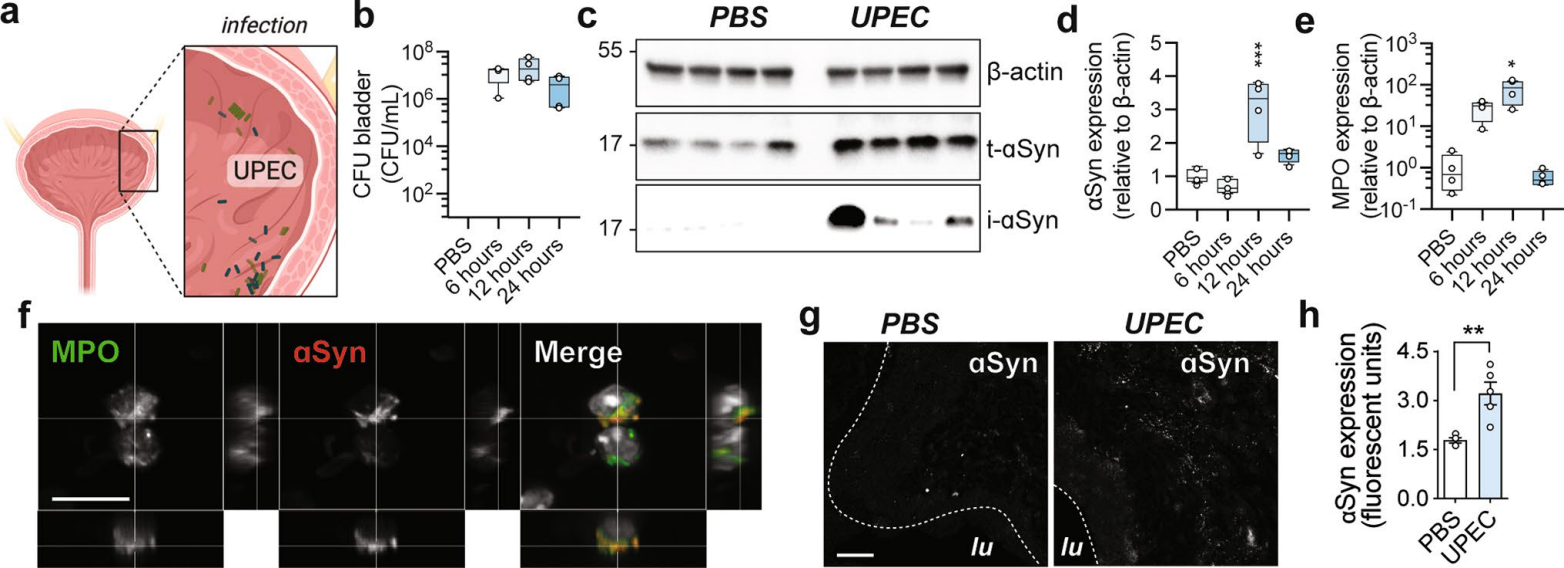
Urinary tract infection triggers endogenous ɑSyn aggregation in vivo. **a** Injection of 10^8^ colony-forming units (CFU) of uropathogenic *E. coli* (UPEC) in the urinary bladder of C57BL/6N mice leads to **b** acute infection with **c** increased expression of total ɑSyn protein (t-ɑSyn) after 12 h of infection. Isolation of insoluble ɑSyn (i-ɑSyn) after sarkosyl treatment of whole urinary bladder homogenates reveals that ɑSyn is aggregated during infection. **d** Quantification of ɑSyn protein expression levels shows a significant upregulation of ɑSyn during infection (*n* = 4, **p < 0.01 with one-way ANOVA and Bonferroni post hoc correction for multiple testing). **e** The expression of ɑSyn coincides with the detection of myeloperoxidase (MPO) protein indicative of neutrophil infiltration (*n* = 4, **p < 0.01 with Kruskal Wallis test and Dunn’s post hoc correction for multiple testing), **f** Confocal Z-stack images of infected mouse urinary bladder shows that neutrophils infiltrating the infected tissue express ɑSyn during infection (scale bar 30 µm). **g** ɑSyn is detected in the lamina propria under basal conditions (lu, lumen, scale bar 50 µm) and increases significantly during infection with UPEC quantified in **h** (*n* = 4, **p < 0.01 with one-way ANOVA and Bonferroni post hoc correction for multiple testing)

Because of the presence of pathological ɑSyn in human urinary bladder and given the putative role of ɑSyn in the immune response, we decided to investigate whether ɑSyn aggregation could be triggered peripherally by an immune-related mechanism. Given the association of UTIs and MSA (Table 1), we chose to use a well-characterized animal model of UTI [45] to study this interaction. Uncomplicated UTIs are caused predominantly by Gram-negative bacteria and the most common causative species is uropathogenic *E. coli* (UPEC), which accounts for approximately 70% of all UTIs [16]. Following infection of WT mice with 10^8^ CFU of UPEC, we assessed endogenous ɑSyn expression during acute infection (Fig. 2a, b). After 12 h of infection, we observe a greater than threefold increase in expression of ɑSyn in urinary bladder via Western blot analysis of whole bladder homogenates (Fig. 2c, d). This increase is also significant after correcting for potential contamination of ɑSyn from erythrocytes, a potential source of ɑSyn which could increase during hemolysis or hemorrhagic inflammation in UTI (Supplementary Fig. 2e, f, online version). At longer time points, the expression of ɑSyn is reduced and in contrast to infection with high titers of UPEC (10^8^ CFU and 10^9^ CFU), infection with 10^7^ CFU yields no significant increase of ɑSyn at 6, 12 or 24 h post-infection (Supplementary Fig. 2e, f, online version). This shows that the increase and kinetics of ɑSyn expression depend on the timing and the strength of the infectious trigger.

Endogenous ɑSyn is expressed in mouse urinary bladder neurons, where it colocalizes with neuronal markers throughout the bladder mucosa and detrusor muscle (Supplementary Fig. 3a–c, online version). During infection, ɑSyn expression coincides with the infiltration of neutrophil leucocytes (Fig. 2e), as indicated by increased levels of myeloperoxidase (MPO) protein expression. Confocal analysis of sections through the wall of infected urinary bladder shows that in addition to neuronal ɑSyn, polynuclear granulocytes positive for the neutrophil marker MPO express ɑSyn (Fig. 2f). Correspondingly, at 12 h after infection, the increase of ɑSyn is significantly accentuated within the lamina propria, quantified in Fig. 2h. Coupled with the fact that we could not detect any observable change in ɑSyn or Pser129-ɑSyn expression in neurons, this suggests that the ɑSyn expression we monitored might originate from the innate immune response.

To further investigate the effect of infection on ɑSyn expression or protein misfolding, we isolated infected urinary bladders to examine the assembly state of ɑSyn. During the earliest steps of infection, neutrophils have an important role as effectors of inflammation in the urinary tract during infection [1]. Neutrophils create an oxidative environment to kill bacteria or other pathogens [36] but oxidative conditions are also known to efficiently catalyze aggregation of ɑSyn [65]. Infected urinary bladders (10^8^ CFU) were isolated at the 12-h timepoint and homogenized under denaturing conditions with 1% sarkosyl. After treatment, we detect aggregated ɑSyn in animals treated with UPEC but not in PBS treated animals (Fig. 2c). This shows that the de novo aggregation of ɑSyn can be triggered in response to a bacterial infection.

### ɑSyn is released as part of neutrophil extracellular traps

**Fig. 3:**
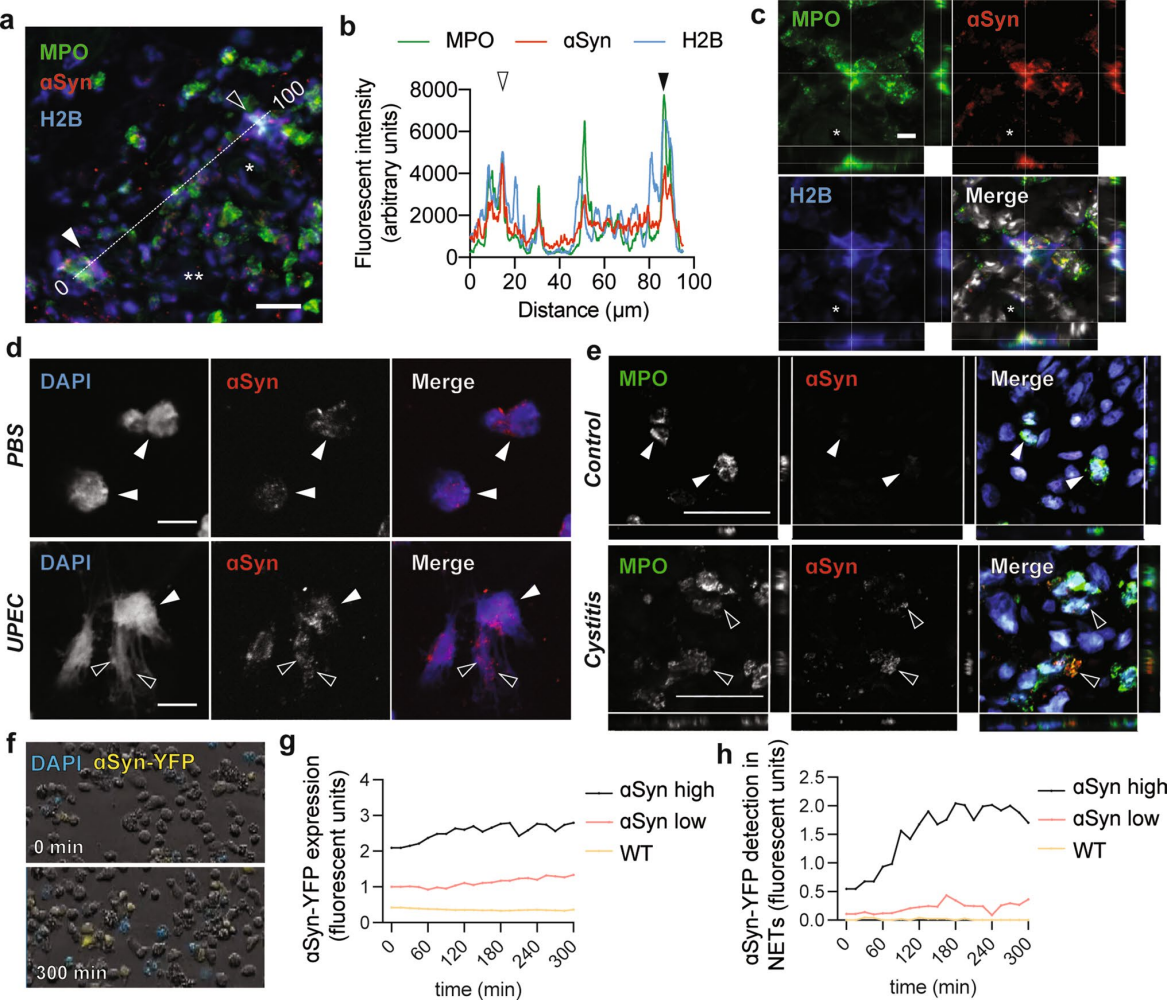
ɑSyn is found extracellularly in neutrophil extracellular traps. **a** Infected urinary bladders at 12 h show NETs with ɑSyn in the vicinity of red blood vessels in the lamina propria (scale bar is 15 µm, * and **indicate two blood vessels). **b** Intensity plot of MPO, H2B and ɑSyn along a 100 µm line in **a** shows overlap between the three markers. Highly ɑSyn expressing areas with strong intensity peaks are observed in NETs (open and closed arrows). **c** Confocal z-stacks of the NET in **a** (closed arrow) shows colocalization between MPO, ɑSyn and H2B (scale bar is 5 µm). **d** Primary human neutrophils express ɑSyn (white arrows), which is released via extracellular traps (black arrows) during infection with UPEC (10:1 MOI, scale bar is 20 µm). **e** In normal human urinary bladder neutrophils express relative low levels of ɑSyn while in a case of cystitis neutrophils with decondensing DNA release ɑSyn extracellularly (black arrows, scale bar is 40 µm). **f** The detection and release of extracellular DNA was visualized via live-imaging in ɑSyn-YFP-expressing dHL-60 stimulated with UPEC (10:1 MOI). Control cells are WT dHL-60 cells that do not express ɑSyn. **g** After infection control cells show background detection of fluorescent signal. ɑSyn-YFP low and high expressing cells show increasing fluorescent ɑSyn-YFP with **h** increased detection of ɑSyn-YFP in extracellular traps (experiment was performed in triplicate and showing one representative experiment)

As a unique microbicidal strategy during host defense, neutrophils can form neutrophil extracellular traps (NETs). NETs are web-like chromatin traps that allow neutrophils to trap pathogens while releasing their enzymatic and reactive intracellular contents [36]. It is well established that ɑSyn is a membrane binding protein that favorably associates with small vesicular organelles in various cell types. Because of its strong association of with granules [5, 38] we tested if infection could trigger the release of ɑSyn from neutrophils during degranulation and whether it thereby could be a source of extracellular ɑSyn that accumulates in peripheral tissue. We examined mouse urinary bladders 12 h after UPEC infection for the presence of ɑSyn in NETs. NETs are formed by DNA, histones and granular proteins and colocalization of these different structures is indicative of NETs [36]. Upon examination of MPO and histones (H2B) with ɑSyn, we detect ɑSyn-positive NETs in urinary bladder in close vicinity to blood vessels of the lamina propria during perivascular extravasation (Fig. 3a). The intensity profiles of the three markers indicate a strong overlap, and the confocal images demonstrate that ɑSyn colocalizes with MPO and H2B (Fig. 3b, c).

Next, we stimulated isolated human primary neutrophils with UPEC, and defined how a bacterial infection influences ɑSyn in neutrophils. Under non-stimulated conditions, ɑSyn is present throughout the cytoplasm of primary human neutrophils (Fig. 3d, upper panel. During infection with UPEC, ɑSyn is detected with the DNA that is extracellular (Fig. 3d, lower panel). Next, we examined the expression of ɑSyn in neutrophils from biopsies of human urinary bladder. We examined the urinary bladder for neutrophil infiltration and ɑSyn expression in tissue from 25 different individuals, including those with cystitis and those with no known underlying condition in the urinary bladder. In normal, non-inflamed human bladders, we detected ɑSyn in neurons, but there was no ɑSyn-immunoreactivity in the sparse circulating neutrophils in the walls of the bladders (Fig. 3e, upper panel and Supplementary Fig. 4a, online version). However, in cases with interstitial cystitis with marked infiltration of neutrophils, we detect expression of ɑSyn in neutrophils in 4 out of 11 cases (Fig. 3e, lower panel and Table 2). In cases with detectable levels of ɑSyn, the expression of ɑSyn was co-localized intra- and extracellularly with DNA or MPO. Neutrophils are known have a very short circulatory lifespan lasting a few hours, but they can be primed at the site of activation, which expands their longevity [36]. The short window in which ɑSyn is expressed during infection in our mouse model and the selective presence of ɑSyn in infiltrating neutrophils of human urinary bladder with cystitis shows that ɑSyn is present and released from neutrophils during inflammatory conditions.

**Table 2:** Cystitis cases examined for ɑSyn expression in urinary bladder

Gender	Type	Category	Infiltrating tumor	Neutrophil infiltration	ɑSyn^+^ neutrophils
Female	Excision	CI	No	−	−
Male	TURB	CI	Surface CIS	−	−
Male	Ureter	–	No	−	−
Male	Bladder	CI	No	+	+
Female	TURB	AI, CI	No	−	−
Male	Cystectomy	AI	No	+	−
Male	TURB	CI	No	+	+
Female	TURB	–	No	−	−
Female	TURB	AI, CI	No	+	−
Female	Excision	–	No	+	−
Male	Excision	CI	No	−	−
Male	TURB	AI, Ci	Extensive	+	−
Male	Excision	–	No	−	−
Female	Excision	–	No	−	−
Female	TURB	AI	No	−	−
Female	Excision	CI	No	−	−
Male	TURB	AI, CI	No	+	−
Male	Excision	AI	No	+	+
Female	Excision	–	No	−	−
Male	TURB	–	No	−	−
Male	TURB	–	No	−	−
Male	Excision	AI	No	+	+
Male	Excision	AI, CI	No	+	−
Male	Cystectomy	–	No	+	−
Male	Excision	AI	No	−	−

Different cases with cystitis were examined for ɑSyn expression in neutrophils

*TURB* transurethral resection of urinary bladder tumor, *AI* acute infection, *CI* chronic infection

The presence of ɑSyn in neutrophils suggests a possible role of ɑSyn in innate immune function. Given the role of ɑSyn in vesicle dynamics [19], we hypothesized that ɑSyn might further facilitate release of NETs. To test this, we differentiated human myeloid leukemia HL-60 cells into mature polynuclear granular leukocytes (dHL-60). dHL-60 granulocytes do not express ɑSyn [55] but by transducing HL-60 cells with lentiviral vectors expressing fluorescently tagged ɑSyn, we can assess the effect of different ɑSyn expression levels on NET function. We generated two stably expressing ɑSyn-YFP cell lines, which expressed the transgene at either low or high levels (twofold increase in ɑSyn-YFP). As a control, we used non-transduced WT dHL-60 cells that do not express ɑSyn. We exposed these cells to UPEC (10:1 MOI) and imaged them at regular intervals over 5 h (Fig. 3f). We observe a significant increase in extracellular DAPI signal, indicative of NETs, and within these NETs corresponding levels of ɑSyn-YFP (Fig. 3g, h). Together, these findings further corroborate that ɑSyn is released from neutrophils during infection.

### Aggregated ɑSyn propagates from the urinary bladder

**Fig. 4:**
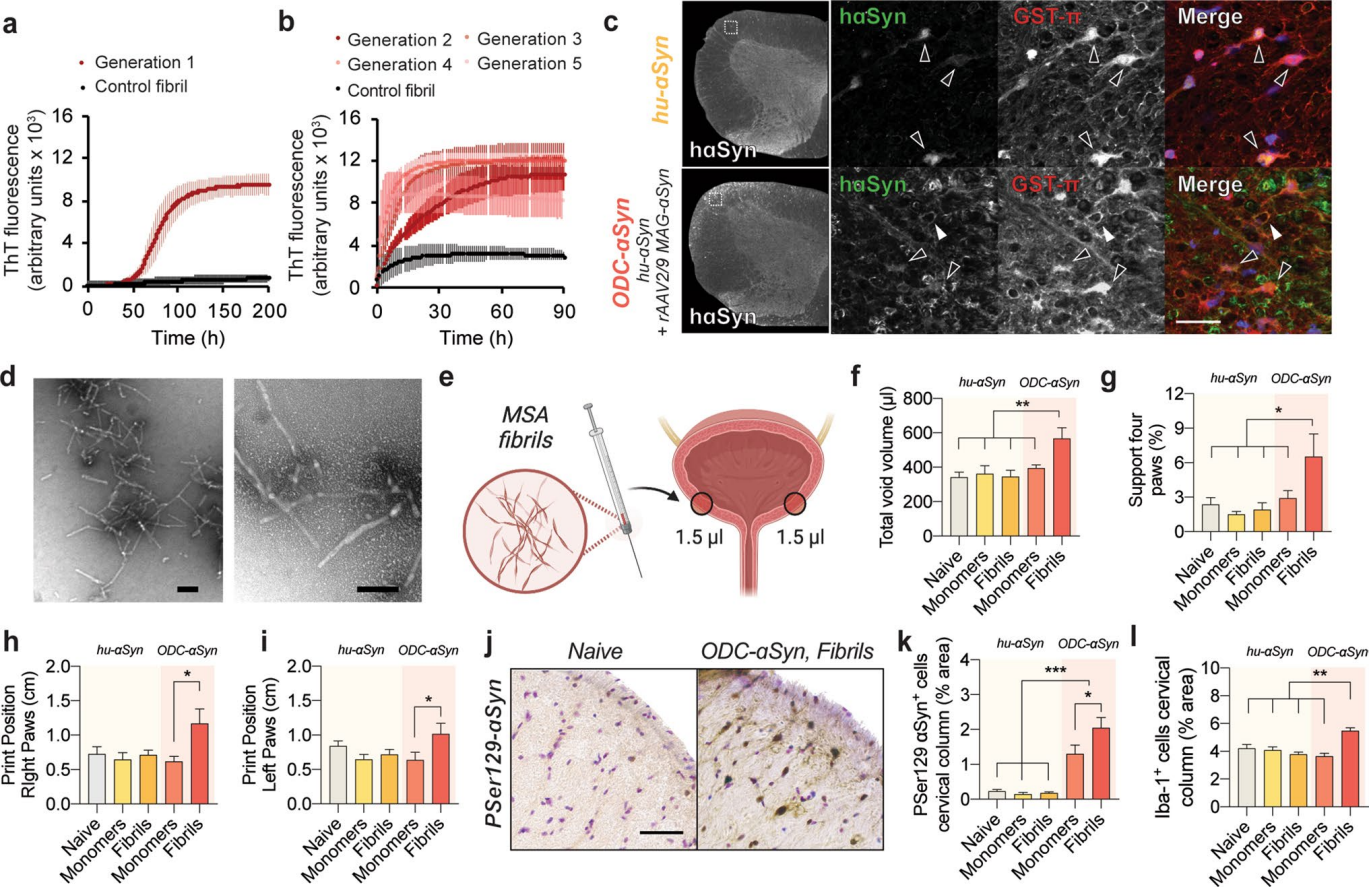
MSA fibrils propagate synucleinopathy from the urinary tract in vivo. To assess peripheral ɑSyn propagation and how oligodendroglial cellular environment can impact the spreading of ɑSyn, ɑSyn fibrils were amplified from human MSA brain. **a** After addition of homogenized and purified MSA brain, ɑSyn monomers were efficiently seeded into fibrillar ɑSyn. **b** Different passages with 5 rounds of amplification generates distinctive MSA fibrils with a distinct ThT profile compared to fibrils generated with recombinant ɑSyn only. **c** Two different animal models are used. Both models express human ɑSyn in spinal cord oligodendrocytes but with differences in expression pattern. Transgenic human ɑSyn mice (hu-ɑSyn mice), express soluble human ɑSyn in cell bodies of oligodendrocytes of the spinal cord (open arrows, upper panel) whereas ODC-ɑSyn mice express human ɑSyn in cell bodies and myelin sheets of mature oligodendrocytes in spinal cord anterior columns (lower panel, closed arrows). **d** Representative images of amplified fibrils of MSA brain recorded by TEM (scale bar 100 nm). **e** Fibrils from MSA brain were injected in two opposite sides of the detrusor muscle of the urinary bladder adjacent to the urethral opening and close the pelvic plexus. After 9 months of injection, animals showed **f** urinary voiding and **g** gait deficits with loss of fine motor control of **h** right and **i** left paws for ODC-ɑSyn mice injected with MSA fibrils but not with monomers (*n* ≥ 6, *p < 0.05, **p < 0.01 with mixed-effects analysis of two-way ANOVA and Tukey post hoc correction for multiple comparison). Motor behavior was measured via automated Catwalk gait analysis. No effects were observed in hu-ɑSyn mice injected with MSA fibrils. **j** Analysis of PSer129-ɑSyn in spinal cord anterior columns and anterior horns reveals that in ODC-ɑSyn mice injection of MSA strains into the urinary bladder leads to **k** increased ɑSyn pathology accompanied by **l** microglial inflammation in the anterior columns (*n* ≥ 6, s.e.m. *p < 0.05, ***p < 0.001 with mixed-effects analysis of two-way ANOVA and Tukey post hoc correction for multiple comparison)

Since we discovered that innate immune cells release ɑSyn and we observed aggregated ɑSyn in urinary bladder tissue, we elected to examine if MSA ɑSyn aggregates can propagate to the CNS. To this end, we first generated MSA ɑSyn fibrils, by isolating ɑSyn fibrils from the cingulate cortex of human MSA brain. Using a sucrose gradient, we isolated ɑSyn from the 10% sucrose fraction and reproducibly amplified ɑSyn fibrils from MSA brains (Fig. 4a, b and Supplementary Fig. 5a–k, online version). Via cyclic amplification, we further amplified the MSA fibrils to remove residual brain material to undetectable levels (Supplementary Fig. 5g, online version).

In MSA patients, glial inclusions are apparent in cervical spinal cord white matter anterior columns including the pyramidal, spinocerebellar and anterolateral fibers [6]. These spinal tracts are among the earliest to be affected in MSA and connect the urogenital nerves and the spinal cord to the midbrain and cerebellum. Since the propagation of ɑSyn in MSA requires the expression of oligodendroglial ɑSyn [52, 62], we used a humanized ɑSyn model (hu-ɑSyn mice) to study MSA progression. Hu-ɑSyn mice lack murine ɑSyn and only express human ɑSyn, at levels comparable to those seen in WT mice [20]. They also express ɑSyn in oligodendrocytes of spinal cord white matter columns already at early age (Fig. 4c). In addition, by performing neonatal intraventricular (IVC) injection with rAAV2/9-MAG-ɑSyn viral vectors [51] in hu-ɑSyn mice, we induced expression of ɑSyn in oligodendrocytes of the spinocerebellar and spino-olivary tracts (ODC-ɑSyn mice, adding to the relevance of the model to MSA, Fig. 4c and supplementary Fig. 6b–e, online version).

We injected 5 µg of MSA fibrils next to the suburothelial plexus of the urinary bladder detrusor muscle bilaterally, and tested if peripheral ɑSyn aggregates in the urinary tract can trigger pathology in the spinal cord akin to that often seen in MSA. In addition, by injecting MSA fibrils in hu-ɑSyn and ODC-ɑSyn mice, we could define if this more permissive environment (expressing ɑSyn in oligodendrocytes) would promote propagation of ɑSyn pathology from the urogenital tract. After urinary bladder injection of MSA fibrils or ɑSyn monomers at 8 weeks of age (Fig. 4d, e), the mice were followed up to 9 months. We examined spontaneous voiding via void spot analysis in freely moving mice [68] and observed that ODC-ɑSyn mice injected with MSA fibrils had a larger voiding volume compared to mice in any of the other experimental conditions tested (Fig. 4f), suggestive of abnormal urinary bladder function. We did not observe any difference in voiding spot frequency (data not shown). In addition, ODC-ɑSyn mice injected with MSA fibrils exhibited impaired gait with postural instability (Fig. 4g) and loss of fine motor control of left and right paws (Fig. 4h, i and Supplementary Fig. 7 a-d, online version) by 9 months (but not earlier, data not shown). These deficits were not apparent in any other groups.

To map histopathological changes, we performed pSer129-ɑSyn immunohistochemistry in sections through the spinal cord. ODC-ɑSyn mice injected with MSA fibrils exhibited increased ɑSyn pathology in white matter cervical and thoracic spinal columns compared to ODC-ɑSyn mice injected with ɑSyn monomers (Fig. 4j, k and Supplementary Fig. 7e–i, online version). While injections of MSA fibrils significantly exacerbated the neuropathology, we also found some pSer129-ɑSyn-immunopositive staining in mice transduced with the ODC-ɑSyn viral vector and subsequently injected with monomers (Fig. 4k). At the cervical and thoracic spinal cord levels of ODC-ɑSyn mice injected with MSA fibrils, the changes in pSer129-ɑSyn staining were accompanied by Iba1-positive microglial activation in ascending anterior columns (Fig. 4l and Supplementary Fig. 8a–i, online version). We did not observe any astrogliosis in any of the conditions tested (Supplementary Fig. 8j–l, online version). Analysis of ɑSyn in urinary bladders injected with different types of ɑSyn assemblies showed faint granular staining of PK-resistant pSer129-ɑSyn with no detectable differences between conditions (Supplementary Fig. 9a, online version). Taken together, we found that injection of MSA fibrils in the urinary bladder of mice overexpressing ɑSyn in oligodendrocytes led to the progressive formation of ɑSyn aggregates, with concomitant signs of neuroinflammation, in the ascending sensory pathways of the spinal cord.

### Urinary tract infections trigger accumulation of ɑSyn in oligodendroglia in vivo

**Fig. 5:**
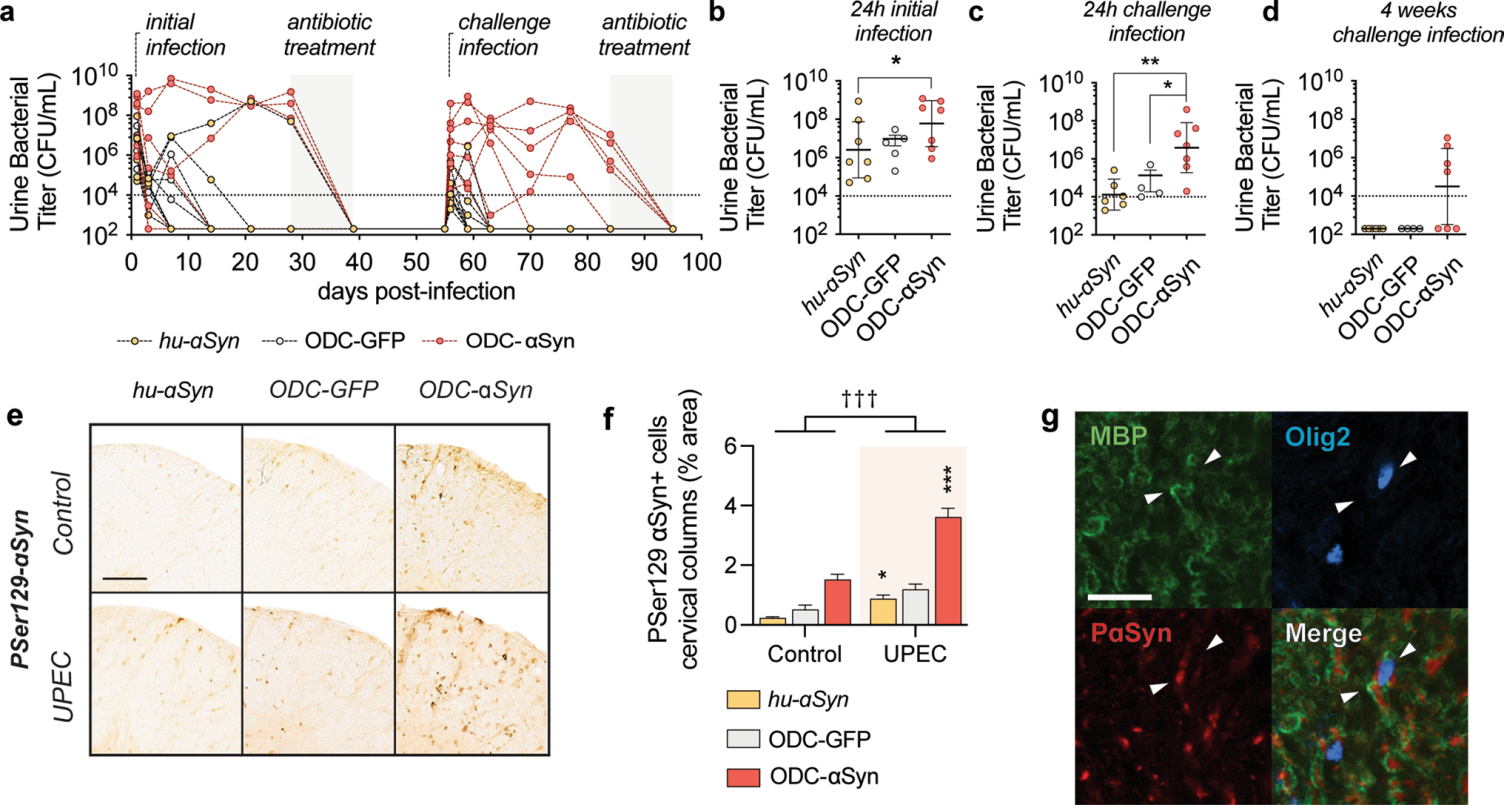
Urinary tract infections promote synucleinopathy in vivo. Different groups of animals representing varying oligodendrogliopathy susceptibilities were infected with UPEC. **a** For the primary infection animals were infected with 10^8^ CFU of UPEC. **b** During acute infection, ODC-ɑSyn mice have higher bacteriuria compared to hu-ɑSyn animals (*p < 0.05 with Kruskal–Wallis analysis and Dunn’s post hoc correction for multiple comparison). After antibiotic treatment and convalescence animals were infected with 10^7^ CFU of UPEC. **c** During challenge infection, ODC-ɑSyn mice have a higher bacterial burden compared to other groups (*p < 0.05, **p < 0.01 with Kruskal–Wallis analysis and Dunn’s post hoc correction for multiple comparison) (**d**) with infection persisting over the 4 following weeks in some animals, indicative of a higher susceptibility towards recurrence in these mice. **e** Nine months after initial infection, animals were analyzed for PSer129-ɑSyn pathology in cervical spinal cord anterior column white matter tracts. **f** In all conditions infected with UPEC higher levels PSer129-ɑSyn were detected in the anterior columns (*n* ≥ 4, s.e.m. *p < 0.05, **p < 0.01 with mixed-effects analysis of two-way ANOVA and Tukey post hoc correction for multiple comparison and ^†††^*p* < 0.001 with mixed-effects analysis of two-way ANOVA between groups). **g** ɑSyn inclusions are present in mature oligodendrocytes of white matter tracts. White arrows indicate a myelinating Olig2/MBP^+^ oligodendrocyte with PSer129-ɑSyn (scale bar 30 µm)

Our epidemiological analysis shows that in the years before diagnosis, MSA patients are more prone to develop recurrent UTIs (OR of 3.76, Table 1). Therefore, we examined if UTIs can exacerbate synucleinopathy in our experimental models using a chronic UTI paradigm [44]. At 7 weeks, animals were infected with 10^8^ CFU of UPEC (primary infection). An additional group, ODC-GFP, was also included to control for any vector-related effects [51]. Four weeks after initial infection, we treated the mice with antibiotics after which they were challenged with 10^7^ CFU of UPEC (challenge infection) (Fig. 5a). Four weeks later, the mice were treated with antibiotics again.

During primary and challenge infections, we observed a trend (not a statistically significant) for higher levels of infectivity in ODC-ɑSyn mice (Fig. 5b, c). In ODC-ɑSyn mice, a chronic infection phenotype was apparent in 4 out of 7 mice after challenge infection, with infectivity persisting up to 4 weeks. To emulate the design of our seeding experiments, we performed behavioral analysis 9 months after the initial infectious trigger, but there were no significant behavioral deficits in any group (data not shown). Despite this, histopathological analysis of the anterior white matter spinal tracts revealed significantly elevated levels of pSer129-ɑSyn in all conditions that had undergone a UTI (Fig. 5d, f). Confocal analysis confirmed that pathological pSer129-ɑSyn immunoreactive deposits accumulate within mature oligodendrocytes (Fig. 5g). Immunohistochemical analysis of urinary bladders at the final time point shows no detectable differences of pathological ɑSyn between groups (Supplementary Fig. 9c, online version). These data show that UTIs can trigger central pSer129-ɑSyn accumulation with oligodendroglial involvement.

## Discussion

The etiopathogenesis of MSA is poorly understood. Genetic studies have examined relatively small numbers of MSA patients and have largely been inconclusive. Similarly, so far no causative environmental factors have been identified. The different neuropathological profiles of the two major forms of MSA (MSA-P and MSA-C), as well as clinical characteristics of the earliest stages of the disease provide important clues to the origins of MSA pathogenesis. Particularly, in MSA-C, there is frequent involvement of spinal cord and lower brain stem. Importantly, MSA patients often report numerous UTIs during the disease prodrome [4, 35]*.* We now provide evidence that a peripheral bacterial infection might act as a trigger of synucleinopathy in MSA, and we present supporting results from epidemiology, human tissues, cell culture and mouse models.

We performed a nationwide case-controlled study examining the association between UTIs and MSA, accessing data from over 5 million people in Denmark. Based on our inclusion criteria, we identified 108 cases diagnosed with MSA. We estimate and hypothesize that the prodrome in MSA is shorter than for Parkinson’s disease (5–20 years). It is uncertain when the first prodromal symptoms of MSA appear, but based on patients initially presenting with REM sleep behavior disorder or autonomic failure and later developing MSA, it has been suggested the MSA prodrome is 2 to 4 years long [4, 35, 59, 67]. Even though the prevalence of MSA is low, and the MSA prodrome relatively short, resulting in a low pretest probability, we found that UTIs associate with MSA diagnosis with a significantly increased OR for MSA 2–8 years after a UTI (in the range of 1.79–3.57). This association did not change after stratifying for sex or after adjusting for urosepsis. Interestingly, it was recently shown that also in people with Parkinson’s disease, UTIs are more common in the 5 years preceding Parkinson diagnosis [10], even though the OR was not as high as that we found for MSA. This hints that there might be overlap between potential triggering mechanisms between both (PD and MSA) these synucleinopathies. Limitations of this case control study include the relatively small number of patients and the lack of clinical data to estimate the duration of preclinical MSA in these particular individuals. While our study cannot prove a causal relationship between UTIs and MSA, it provides tentative support for the idea that UTIs can trigger a synucleinopathy that presents as MSA several years later.

The etiopathogenesis of UTIs differ between females and males for several reasons. In both sexes, transmucosal viral or bacterial infection from the rectum or retrograde urethral infection are common causes [58]. It is believed that most cases of lower male UTIs develop and resolve asymptomatically, leading to underestimation of male UTI frequency [43, 64]. Older men can develop structural and functional urological abnormalities such as urethral stricture, bladder stones or prostatic hyperplasia, leading to recurrent UTIs with sometimes life-threatening infections [58, 64]. Other exclusively male conditions, such as prostatitis could conceivably trigger particularly vigorous immune and inflammatory responses. It is conceivable that this increases the likelihood of aberrant ⍺Syn involvement and as a consequence affects the male-to-female distribution of MSA. Similarly, due to shared innervation and sensory pathways (involving in the lower sacral segments of the spinal cord) between the urogenital system, colon, rectum and other visceral and non-visceral barrier sites such as the skin, infections at any of these sites might all influence the risk of MSA [9, 17, 23]. Indications such as prostatitis, nephritis, cystitis, urethritis and related codes are included in our analysis (Supplementary table 1, online version). In short, we believe that factors such as an underestimation of UTIs in men, relatively high likelihood of severe genitourinary infections in men, the extensive cross-organ innervation and infections that might occur at multiple sites, can all contribute to the observed balanced male-to-female distribution in MSA.

We examined urinary bladders obtained postmortem from MSA patients and age-matched controls and found numerous aggregated and insoluble PSer129-ɑSyn deposits. Deposits were present throughout the tissue, most densely around blood vessels. Next to its expression in neurons, native ɑSyn is also present in vascular endothelium, smooth muscle cells [34, 61] and circulating immune cells [22]. The transcriptome of peripheral immune cells of MSA patients are consistent with a response to infection [53, 54]. The aggregated nature of ɑSyn in the bladder nerves and around blood vessels might suggest an ongoing inflammatory condition. Furthermore, the dense perivascular distribution of ɑSyn raises the possibility that the de novo aggregation of ɑSyn is not seeded exclusively by neuronal ɑSyn, but that immune cells might be another source.

Given that ɑSyn plays a role the immune system [26], that UTIs are common in the general population [16], that we found a strong association between UTIs and MSA and that a high proportion of MSA patients eventually develop chronic UTIs [49], we decided to explore if a UTI can trigger ɑSyn expression and aggregation. We used a well-characterized mouse model of UTI in WT mice and found that ɑSyn expression significantly increases in the urinary bladder during acute infection, and that insoluble ɑSyn aggregates form. This response was concurrent with inflammation and infiltration of activated innate immune cells. Notably, we observed extravasating neutrophils in the bladder walls with NETs that were immunoreactive for ɑSyn.

In parallel to these observations, we found that ɑSyn is expressed in primary human neutrophils and in neutrophils present in the wall of human urinary bladder. In vitro stimulation of human neutrophils with UPEC caused the release of ɑSyn and also in biopsies from acute interstitial cystitis, we detected ɑSyn-immunoreactivity in and around neutrophils. Since ɑSyn is present in NETs, we investigated the role of ɑSyn during NETosis. Via live cell imaging of fluorescently labeled ɑSyn, we observed that with increasing levels of ɑSyn, dHL-60 cells more efficiently release NETs and ɑSyn was also present within these extracellular traps. We focused our attention on the expression of ɑSyn in neutrophils, but other types of granulocytes, as well as macrophages and lymphocytes, are also known to express ɑSyn [22]. Possibly these immune cells contribute to changes in ɑSyn levels in the infected urinary bladder, and they deserve further future investigation. However, these immune cells are not known to release intracellular content (and ɑSyn) like neutrophils.

We and others have suggested that ɑSyn aggregation might commonly occur in peripheral tissues [30, 40, 60, 63], and only when the host is susceptible (e.g., due to genetic predisposition or aging) will the aggregated ɑSyn propagate to the CNS. Consistent with this idea, we detected ɑSyn deposits in urinary bladder not only of people with MSA, but also in control subjects with no history of neurodegenerative disease. Early appearance of urinary and erectile dysfunction is common in MSA and it has been proposed that they might represent a specific subtype [47, 56]. Classically, patients develop urinary retention with the appearance of neurological signs. However, some MSA patients experience urogenital symptoms before motor deficits [4, 35]. They typically exhibit urinary urgency and incontinence, followed by urinary retention, erectile dysfunction, anorgasmia or gut symptoms suggesting the proximal involvement of the lower spinal cord or infrasacral regions [47].

Several studies have shown that peripheral injection of fibrillar ɑSyn can trigger neuropathology in wild-type and transgenic animals via seeded aggregation, but these models have lacked oligodendroglial pathology that mimics MSA [8, 33]. To test if aggregated ɑSyn can spread from the urogenital tract to the CNS we injected ɑSyn fibrils, extracted and amplified from human MSA brain, into the urinary bladder of hu-ɑSyn and OCD ɑSyn mice. These mice express ɑSyn in oligodendrocytes of the spinal cord and therefore are likely more prone to develop oligodendrogliopathy. Nine months after injection of MSA fibrils into the wall of the urinary bladder, we observed pathological changes in the spinal cord of OCD-ɑSyn mice. Furthermore, the mice developed changes in urinary bladder function with changes in voiding volume. These changes were not accompanied with changes in voiding frequency but the injection of ɑSyn assemblies injected next to the pelvic ganglia might have possibly resulted in more local pathology in peripheral nerves, in contrast to more widespread CNS pathology that is seen in later stages of MSA. This could have led to an increase in voiding volume instead increased voiding frequency. Next to bladder deficits, we observed gait abnormalities. These changes were absent in the hu-ɑSyn mice and injection of non-aggregated, monomeric ɑSyn in the urinary bladder did not lead to pathology in any of the paradigms. The reason for the absence of pathology in hu-ɑSyn mice injected with MSA fibrils could be multifold. Possibly, in this paradigm, the host environment is favorable for the propagation of ɑSyn or alternatively, there was not enough time to allow the pathology to propagate after urinary bladder injection of MSA fibrils. For MSA fibrils to propagate in vivo, ɑSyn seeds have to amplify with monomeric ɑSyn in oligodendrocytes [52]. Therefore, monomeric ɑSyn has to be expressed within oligodendrocytes since templated amplification of MSA strains cannot occur otherwise. Under normal conditions, expression of ɑSyn in oligodendrocytes is low, but recent reports have shown that oligodendroglial expression of ɑSyn can increase in response to inflammatory insults [14, 28, 39] and during aging [32]. The ODC-ɑSyn mouse model we used might mimic such a disease-associated environment, making them more permissive to the development of MSA.

We postulated that repeated UTIs could trigger central pathology and we performed infections using the same experimental models. During primary and challenge infection, ODC-ɑSyn mice had a significantly higher infectious burden compared to other conditions and there was a trend for these mice to develop chronic UTIs. A switch from self-limiting to chronic UTIs is regulated via immune checkpoints [25]. The pro-inflammatory cytokine TNFɑ can strongly influence susceptibility to recurring UTIs [69] and its sustained elevation can alter the immune response by prolonging and worsening its course. Levels of pro-inflammatory TNFɑ are inversely correlated with disease severity in MSA and are highest during early stages [31]. Given that MSA patients are at higher risk to develop recurring UTIs already during the prodrome it could suggest a bidirectional relationship between peripheral infections and disease pathogenesis by triggering an aggravated disease onset. Using our repeated UTI paradigm, we find that infections impacted synucleinopathy across groups with consistently higher levels of Pser129-ɑSyn in oligodendrocytes in the CNS. Taken together, this shows that infections potentially can impact different stages of the disease.

In this study we used epidemiology to identify a novel association between MSA and UTIs several years earlier, and we performed a series of human histopathology, cell culture and mouse experiments that provide support for the idea that a bacterial infection in a peripheral organ can trigger of synucleinopathy, with innate immune cells playing a central role. We present a mouse model in which the injection of MSA fibrils into the urinary bladder can cause behavioral deficits and spinal cord pathology with some cellular and anatomical features in common with MSA. In the same experimental mouse models, repeated UTIs can trigger central pathology with oligodendroglial involvement. These findings, coupled with numerous reports of urogenital dysfunction in the MSA prodrome, suggest that in some cases of MSA the disease process might start in the urogenital region.

A limitation of this study is the relatively low number of diagnosed cases with MSA for epidemiology and the lack of clinical data to accurately define the prodromal phase. To establish a clear role for ɑSyn during host–pathogen interactions, future studies should explore if ɑSyn pathology during infection is specific for UTIs, the conditions in which an immune-triggered response can propagate to the brain and if even longer survival times leads to greater spread of ɑSyn pathology (also to the brain). This will facilitate the identification of early changes in the urogenital organs that can be targeted in future MSA therapies.

## Materials and methods

### Epidemiology

We included all adults (18 years of age or older) registered as living in Denmark between 1 January 2016 and 31 December 2018. Multiple system atrophy (MSA) was defined as a registration in the Danish National Patient Register (DNPR) with a confirmed diagnosis with ICD-10 code G23.2 (Multiple system atrophy, parkinsonian type) and G23.3 (Multiple system atrophy, cerebellar type). For each case, we randomly sampled 10 controls among all potential persons in the background population with same sex, birthdate ± 90 days, and cohabitation status. Cohabitation status was defined as single or living together defined as two adults living at the same address and are married, in registered partnership, having children together, or with an age difference lower than 15 years.

Urinary tract infections (UTIs) were defined as a registration in DNPR with a main or secondary diagnosis of UTI, a redemption of drugs only approved for treatment of UTI or a redemption of antibiotics labeled with UTI as indication in the Register of Medicinal Product Statistics. ICD-10 codes of selected diagnoses and ATC-codes and labels are presented in Supplementary table 1. Any redemption within 14 days of treatment termination of an UTI (based on redemption date and daily defined dose) is considered as prolonged treatment of the same UTI. Recurrent UTI was defined as two or more UTI within 6 months or three or more UTI within a year.

To elucidate the effect on MSA risk of UTIs at different time periods before first diagnosis of MSA, we calculated odds ratios (OR) with corresponding 95% confidence intervals (95% CI). An association of UTIs in the period 2–8 years was chosen to increase prodromal MSA probability. To avoid reverse causality, we did not count UTIs in the 2 years prior MSA onset. The period was split into two prodromal intervals, 2–4 years and 5–8 years prior the first diagnosis of MSA. We also analyzed the effect of a recurrent UTI. Analyses were carried out using SAS software version 9.4 (SAS Institute Inc, Cary, NC). All *p* values were 2-tailed with the significance level set at *p* < 0.05.

### Animals

We utilized 7- to 8-week-old C57BL6J or C57BL6N wild-type (WT) animals that were bred in the vivarium at the Van Andel Institute for infections with UPEC. For MSA fibril injections, we used transgenic human ɑSyn (hu-ɑSyn) mice obtained from Jackson Laboratories that express the full human ɑSyn sequence, including the human ɑSyn promoter (Tg(SNCA)1Nbm) [20] or Snca^−^; PAC-Tg(SNCA^WT^), stock No 010710. Transgenic hu-ɑSyn mice were P0 for intracerebroventricular viral vector injection and 8 weeks old for fibril injection. Mice were housed at a maximum of 4 per cage under a 12-h light-/dark cycle with ad libitum access to water and food. The housing of the animals and all procedures were carried out in accordance with the Guide for the Care and Use of Laboratory Animals (United States National Institutes of Health) and were approved by the Van Andel Institute’s Institutional Animal Care and Use Committee (IACUC, AUP 17-11-019).

### Patients

Paraffin-embedded urinary bladder tissue from MSA subjects and age-matched control subjects were obtained from the Banner Sun Health Research Institute, Sun City, AZ (Supplementary Table 2). All MSA cases were examined and confirmed for oligodendroglial ɑSyn pathology. Paraffin-embedded cystitis cases and age-matched control cases were obtained from the Pathology and Biorepository Core at the Van Andel Institute, Grand Rapids, MI. Tissue used in this study was collected with the informed consent of the patients. Protocols were reviewed and approved by the Banner Health and Van Andel Institute Institutional Review boards.

### UPEC infections

The UPEC isolates used in this study were the human isolates UTI89 and its kanamycin-resistant isolate UTI89 attHK022::COMGFP (kanamycin-resistant UPEC). Strains were picked with an inoculation loop from a frozen glycerol stock and cultured statically for a first overnight passage at 37 °C in 20 mL Luria–Bertani broth (LB). UPEC were subsequently subcultured 1:1000 in fresh 20 mL LB statically for 18 h to induce type 1 pilus expression. The resulting cultures were centrifuged at 3200×*g* for 12 min, resuspended in 10 mL sterile PBS and diluted to approximately 2 × 10^9^ colony-forming units (CFU)/mL (OD_600_ = 0.22) or 1 × 10^8^ CFU/50 µL for infection. For dose–response experiments, all cultures were diluted to 1 × 10^8^ CFU per 50 µL of inoculum. For dose response applications, UPEC was diluted to 1 × 10^8^ or 1 × 10^7^ CFU per 50 µL or concentrated to 1 × 10^9^ CFU per 50 µL inoculum. For challenge infection, 1 × 10^7^ CFU/50 µL were injected. Bacteria were inoculated into the urinary bladder of female mice via transurethral catheterization under isoflurane anesthesia as previously described [24]. To monitor infection outcome and urinary bladder bacterial infectious titer, urinary bladders were aseptically isolated at the indicated time point and homogenized with a tissue homogenizer (Omni tissue homogenizer, TH) for 20 s in 1 mL sterile PBS. Homogenates were serially diluted in PBS and 50 µL of each dilution was spotted on LB agar plates to assess for total number of CFU from urinary bladder homogenates after infection. For urine bacterial titers, 10 µL of infected urine was serially diluted in PBS and spotted on LB agar plates to assess the total number of CFUs. To stop infection during chronic infections, animals were treated with trimethoprim/sulfamethoxazole at a concentration of 270 and 54 μg/mL in drinking water for 10 days. Antibiotics were changed every other day.

#### Live imaging

HL-60 cells were cultured in suspension in RPMI 1640 medium with 20% fetal bovine serum (FBS) at 50 I.U./mL. Cells were cultured in T-75 Corning flasks with vented caps (Cat. No. 431080) and stored at 37 °C in 5% CO_2_. Confluency of the cells was maintained between 500,000 to 1,000,000 cells/mL. Cell counts were accomplished using trypan blue on a Bio-Rad automated cell counter T-10. Media was changed every 3 days by transferring the culture to an Eppendorf tube, spinning the cells down at 300×*g* for 4 min, and resuspending cells in new media. Cells were differentiated by culturing cells in media with 1 μM all-trans retinoic acid (ATRA) dissolved in DMSO for 5 days. The ɑSyn-YFP-expressing HL-60 cell were generated by stable lentiviral transduction of HL-60 cells with LV CMV-ɑSyn-YFP at three different doses. Cells were FACS-sorted into low and high ɑSyn-YFP-expressing cells, based on YFP intensity. After differentiation with ATRA for 5 days, we challenged HL-60 WT, low-ɑSyn-YFP and high-ɑSyn-YFP with UPEC at a ratio of 10 UPEC to 1 HL-60 cell and imaged for 400 min using a Carl Zeiss Celldiscoverer 7, taking a picture every 15 min of a 6 by 6 field. The 6 by 6 field was captured using a 20× objective and was digitally amplified by 2×. The videos were run at a speed of 2 frames per second.

### Quantification of in vitro NETs specific fluorescence

In vitro NETs’ specific fluorescence was quantified, offline, with a custom data stream processing script in Bonsai v2.3.0. utilizing only open source packages and their native functions within. First, the color model for time-lapse videos was converted, post hoc, to HSV (Hue/Saturation/Value) to minimize subtle effects of lighting variability on object detection. Next, fluorescent color channels were isolated by HSV threshold values with the HSV threshold function and NETs specific fluorescence was further isolated using contour detection. This resulted in the quantification of NET-like cellular events, as well as the total fluorescence signal for each color channel. Once the only remaining pixel values in the camera’s field of view were restricted to NETs events and excluding apoptotic cells (Supplementary movie 1), this value was quantified as the average values of all pixels in the camera’s field of view at a given time point. Finally, the fluorescence values were normalized as a % across all aSyn groups [41].

### Viral vector production

Recombinant adeno-associated viral (rAAV) vector were generated as described previously [66]. Briefly, production plasmids include the rAAV 2/9 serotype construct, the AAV transfer plasmid encoding human ɑSyn under the control of the oligodendroglial specific promoter myelin-associated glycoprotein (MAG) and the pAdvDeltaF6 adenoviral helper plasmid. The MAG promoter was generated by amplification of the MAG promoter from HeLa cells (Supplementary Table 3). rAAV2/9-MAG ɑSyn was produced in Hyperflasks seeded with HEK293T cells (ATCC, Manassas, VA, USA) in Opti-MEM (Invitrogen, Merelbeke, Belgium) without addition of serum. The supernatant was collected 3 days after producer cell transfection and concentrated with tangential flow filtration. Viral vectors were subsequently concentrated via iodixanol step gradient and centrifugation at 27,000×*g* for 2 h. Gradient fractions were collected and pooled between refraction index 1.39 and 1.42 after which a final centrifugation and concentration step was performed with Vivaspin 6 columns (PES, 100,000 MWCO, Sartorius AG, Goettingen, Germany) at 3000×*g*. Samples were aliquoted and stored at − 80 °C for further use. Viral titers were determined via real-time PCR using a primer probe set for polyA (Supplementary Table 3) to assess genomic copies (GCs) and were approximately 6 × 10^12^ GC/mL.

### Intracerebroventricular injections

Newborn pups were injected intracerebroventricularly (ICV) at P0 (6–12 h after birth) and when milk spots were visible. Only half of the pups were taken from their mother during nursing. Before surgery, pups were cryoanesthetized on a metal plate on ice to cool down body temperature to 4 °C and subsequently transferred to a cooled neonatal surgery frame that was maintained at 2–6 °C with dry ice. The head was wiped with 70% ethanol, leveled parallel with the injection frame and fixed gently between two ear bars. Cranial blood vessels were used to determine the lambda sutures for ICV injection coordinates. A 34G 10 µl Hamilton needle (Hamilton, Reno, NV, USA) was placed at A/P: + 0.165 cm and M/L: ± 0.080 cm from lambda. The bevel of the needle was used to pierce the skull and positioned just below the flattened skull after which it was lowered to D/V: − 0.170 cm and retracted to D/V: − 0.130 cm for ventricular injection. Injection was performed bilaterally for a volume of 2 µL for each ventricle (for a total volume of 4 µL) at a perfusion rate of 0.5 µL/min and a total titer of approximately 2 × 10^10^ GC. After the injection, the skull was flattened by gently retracting the needle to open the ventricles and the needle was left for another 2 min to allow the injected bolus to spread throughout the ventricles. Viral vector was supplemented with 0.5% trypan blue dye to achieve a 0.05% trypan blue solution in order to assess successful ventricular injection. After injection, pups were allowed to recover on a 37 °C heating pad and returned to their home cage. The procedures as described above for IVC surgery were approved by the Van Andel Institute Institutional Animal Care and Use Committee (IACUC).

### Human disease brain tissue for fibril amplification

Frozen brain tissue from the anterior cingulate was obtained from subjects with MSA, and age-matched control subjects from the Michigan Brain Bank (University of Michigan, Ann Arbor, MI, USA). Brain tissue was collected with the informed consent of the patients. Protocols were approved by the Institutional Review Board of the University of Michigan and abide by the Declaration of Helsinki principles. Samples were examined at autopsy by neuropathologists for diagnosis.

### Brain tissue processing

Total of 600 μL lysis buffer (1xPBS, 2 mM EGTA, 2 mM EDTA, PhosSTOP™ (cat# 4906845001, Sigma-Aldrich, Burlington, MA, USA), cOmplete™ EDTA-free Protease Inhibitor Cocktail mini (cat# 11836170001, Sigma-Aldrich), 6 μL/mL saturated phenylmethylsulfonyl fluoride (PMSF), and 1 mM sodium azide in PBS) was added to 0.5 g of brain tissue (anterior cingulate) of individuals diagnosed with MSA, and age-matched controls without αSyn pathology (Supplementary Table 4). Tissue samples were homogenized with 3.2 mm stainless steel beads three times for 1 min (speed 4) with cooling on ice for 5 min in between processing (Nova Advance homogenizer, Next Advance, Troy, NY, USA). Then 300 μL of lysis buffer was added, and samples were homogenized one more time for 2 min.

Homogenized samples were centrifuged at 1000×*g* for 10 min. Then the supernatant was additionally centrifuged at 18,000×*g* for 10 min. TritonX-100 was added to the resulting supernatant to make a 2% final concentration. After incubation with rocking for 30 min at 4 °C, samples were centrifuged at 18,000×*g* for 10 min. Then the supernatant was loaded on the top of a sucrose gradient. Gradients were prepared by layering 40% (w/v), 30% (w/v), 20% (w/v), 15% (w/v), 10% (w/v), and 5% (w/v) sucrose in PBS. Starting with the highest concentration, sucrose solutions were added one at a time followed by flash freezing in a liquid nitrogen. Gradients were centrifuged at 200,000×*g* (SW 41Ti, Beckman Coulter) for 16 h at 4 °C. Gradient layers corresponding to different sucrose concentrations were pooled and consequently used to seed fibril formation of recombinant αSyn.

### Recombinant protein preparation

Recombinantly expressed full-length αSyn was prepared as previously described. Briefly, E. coli cell cultures were grown in LB media containing ampicillin and induced with 0.5 mM isopropyl β-d-1-thiogalactopyranoside for 3 h. Cells were harvested by centrifugation, the cell pellet then resuspended in lysis buffer (200 mM Tris–HCl pH 8.0, cOmplete™ EDTA-free Protease Inhibitor Cocktail mini (cat# 11836170001, Sigma-Aldrich/Roche, Basel, Switzerland), and 6 μL/mL saturated PMSF) and cells were lysed using an Emulsiflex homogenizer (Avestin, Ottawa, Canada). After centrifugation for 15 min at 32,000 g, 0.23 g/mL ammonium sulfate was added to the supernatant. After incubation for 20 min, samples were centrifuged 32,000×*g* for 20 min. The pellet was resuspended in 20 mM Tris, pH 8.0 (Buffer A), and dialyzed against 4 L of buffer A, which was exchanged twice. The next day, samples were loaded onto a 5 mL Q-Sepharose FF column (GE Healthcare) equilibrated with Buffer A and eluted against a linear gradient of Buffer B (1 M NaCl, 20 mM Tris–HCl, pH 8.0, pH 8.0). Fractions containing αSyn were identified using SDS-PAGE, collected, and further purified by pushing the protein through 50 kDa Amicon Ultra-15 Centrifugal Filter Unit (Millipore Sigma, Burlington, MA). The flow through was further concentrated and assessed by SDS-PAGE. Endotoxins were removed by incubation of purified αSyn with Pierce High-Capacity Endotoxin Removal Resin (Cat. # 88270, Fisher), as specified by the manufacturer. Levels of endotoxins were lower than 0.001 Endotoxin units/µL.

### Fibril formation

Fibril assays were carried out with 100 μM αSyn in a sample buffer containing 150 mM NaCl, 25 mM sodium phosphate pH 7.5, and 1 mM sodium azide. Prior to the assay recombinant αSyn was filtered through a 0.05 μm pore filter. The first generation of fibrils was formed by adding 2% w/w (protein weight) of fractionated brain MSA tissue pulled from 10% sucrose gradient. The progeny fibrils (second, third, fourth, etc. generations) were produced by adding 15% w/w (protein weight) of sonicated parent fibrils to the monomeric αSyn. Volumes of 60 μL were dispensed in each well of 384-well glass bottom plates (Greinier, Monroe, NC, USA, cat# 781892). Plates were then incubated at 37 °C in FLUOstar Omega (BMG Labtech Inc, Ortenburg, Germany) by shaking. Three wells were supplemented with 10 μM ThioflavinT (ThT) to monitor the progress of fibril formation. Fluorescence was measured with gain set at 90%, an excitation wavelength of 440 nm and emission wavelength of 490 nm. At reaction completion (ThT fluorescence reached plateau), samples from all wells, except for the ones containing ThT, were combined.

### Preparation of fibrils for animal injection

Fibrils of αSyn were washed to remove unpolymerized protein. After spinning 1 mL of sample at 17,000×*g* for 5 min, the pelleted fibrils were resuspended with a sample buffer without sodium azide. Sample spinning/resuspension was repeated twice. The last pellet was resuspended with a 200 µL sample buffer without sodium azide, resulting in 5× concentrated fibrils. After testing for endotoxin using (Pierce LAL Chromogenic Endotoxin Quantitation Kit, cat# 88282), fibrils were sonicated. The concentration of αSyn (monomer) was determined by dilution of the sample 5× with 6 M guanidinium hydrochloride, followed by boiling for 1 min. After cooling, protein concentration was determined by measuring A_280_ with an extinction coefficient of 5960 M^−1^ cm^−1^. Samples (350 µM monomeric αSyn) were flash frozen in 25 µL aliquots on a liquid nitrogen and stored at − 80 °C until use.

### Sonication

If specified in the test, fibrils were sonicated (PIP 50, DF 10%, and CPB 200) for 120–160 cycles (1 s ON and 1 s OFF) in tubes with disposable probes (microTUBE-130 AFA Fiber Screw-Cap, cat# PN 520216, Covaris, Woburn, MA, USA) in an M220 Focused-ultrasonicator (Covaris).

### Transmission electron microscopy (TEM)

Negatively stained specimens for TEM were prepared by applying 5 μL of sample to hydrophilic 400 mesh carbon-coated Formvar support films mounted on copper grids (Ted Pella, Inc., Redding, CA, USA, cat# 01702-F). The samples were allowed to adhere for 4 min, rinsed twice with distilled water, and stained for 60–90 s with 5 μL of 1% uranyl acetate (Ted Pella, Inc.). All samples were imaged at an accelerating voltage of 80 kV in a JEM-1400 (JOEL).

### Urinary bladder recombinant protein injections

Male and female transgenic 8-week-old transgenic hu-ɑSyn animals were anesthetized with a mixture of oxygen and isoflurane. After disinfection of the lower abdominal region, a small incision was made above the urinary bladder after which the urinary bladder was isolated and fixed for injection. Mice were inoculated with 1.5 µL of MSA fibrils or ɑSyn monomers at equivalent concentrations of 250 µM for a total of 3 µg in two opposite spots of the lower detrusor muscle adjacent to the urinary tract. Amplified fibrils from 3 MSA patients were mixed and injected as one bolus. Each injection was performed with a microinjector and confirmed with 0.05% toluidine blue to visually assess correct injection and spreading of the injected material throughout the urinary bladder tissue. After injection, the lower abdominal peritoneum and skin were sutured and disinfected again. Animals were allowed to recover on a heating pad at 37 °C and monitored during recovery.

### Behavioral analysis

To assess urinary bladder function and voiding, the spontaneous void spot assay was conducted on freely moving animals, as described previously [68]. Mice were transferred to a clean, empty cage with a Whatman Grade 540 hardened Ashless filter paper covering the cage bottom with nestlet enrichment placed on top of the membrane. One mouse was housed per cage during the voiding analysis. The assay was conducted over a 4-h period on male mice in the morning from 9 AM until 1 PM. Animals had access to food but not to water and were tested in the same location as they were normally housed. Filter papers were imaged with UV light and analyzed with Fiji software (1.46). A standard was calculated to measure total voiding volume and voiding spot frequency.

Gait analysis was performed using the Catwalk XT automated gait analysis system that consists of a 130 × 10 cm glass-floor walkway with fluorescence light beaming in the glass to illuminates the animal’s paws (Noldus Information Technology). Each experiment was performed according to the manufacturer’s instructions. Briefly, mice were acclimatized for 1 h in the behavior room with ambient red lighting. To encourage runs across the catwalk the home cage of each mice was used as cue. The pawprint detection settings were determined automatically by the catwalk XT dedicated software (ver. 10.6) by tracking a control mouse with a weight representable of the cohort. All mice were tested in 3 consecutive runs over 3 consecutive days at 6- and 9 months post-inoculation with MSA fibrils or monomeric ɑSyn protein. Compliant runs were defined as lasting between 1 and 5 s with a maximum speed variation of 60% [7]. If a subject failed to run across the catwalk, a run was repeated until compliant. In rare instances, a mouse completed < 3 compliant runs. All runs were recorded and pawprints labeled automatically. Acquired data were segmented according to strict inclusion criteria including a minimum number of steps per run of 10, average speed ranging from 1 to 50 cm/s while all non-compliant or partially characterized runs were excluded. Accordingly, 81.6 ± 6% of acquired runs were included for further data analysis and graphic representation (1177/1457 runs). For all animal behavioral experiments, observers were blinded to the experimental conditions during assessment and analysis.

### Euthanasia and tissue collection

Animals were anesthetized with sodium pentobarbital (130 mg/kg; Sigma-Aldrich) for tissue collection at the indicated time points. Urinary bladders were isolated prior to perfusion and homogenized or fixed with 4% PFA with overnight fixation for subsequent protein analysis or histological analysis, respectively. For animals injected with recombinant protein, perfusion was performed at the 9-month time point (when animals were approximately 11 months old) with isotonic saline. The urinary bladder was isolated before perfusion and was fixed via 4% PFA overnight fixation. Spinal cord and brain were isolated after transcardial perfusion with saline and fixation with 4% PFA. Both spinal cord and brain were post-fixed overnight with 4% PFA and then stored at 4 °C in 30% sucrose in phosphate buffer until sectioning.

### Western blotting

Urinary bladders were isolated from infected animals in 1 mL sterile PBS supplemented with protease and phosphatase inhibitor (Halt Protease Inhibitor Cocktail 100×, ThermoFisher) and homogenized using a tissue homogenizer (Omni tissue homogenizer) for 20 s. Samples were spun at 6000×*g* at 4 °C for 5 min to remove cell debris. The supernatant was heated at 95 °C for 10 min with Laemmli buffer and stored at − 80 °C for downstream analysis. For detection of aggregated ɑSyn, urinary bladders were homogenized in sterile PBS with protease and phosphatase inhibitor. Homogenized tissue was treated with 1% sarkosyl for 60 min at room temperature while gently rotating. Denatured samples were spun at 6000×*g* for 10 min at 20 °C to remove remaining cell debris and the supernatant was used for isolation of aggregated protein. Sarkosyl samples were ultracentrifuged at 100,000×*g* for 60 min at 20 °C. The resulting pellet was washed with PBS and resolubilized in PBS with 1% sarkosyl for a second round of ultracentrifugation for 20 min at 20 °C. The pellet was washed in Laemmli buffer containing 2% SDS to disaggregate ɑSyn assemblies and denatured at 95 °C for 10 min. Samples were stored at − 80 °C for downstream analysis. Samples were separated via SDS-PAGE using a 4–15% Criterion TGX precast gels (Bio-Rad) and transferred using the Trans-blot Turbo system (Biorad) to a PVDF membrane. Membrane blocking was performed with 5% BSA in 0.1% triton-X in PBS for 30 min whereafter primary antibody was applied according to the concentration listed in Supplementary Table 5 for O/N incubation at 4 °C in 5% BSA in 0.1% triton-X in PBS. Membranes were washed three times next day with 0.1% triton-X in PBS, and incubated with HRP-labeled secondary antibody (Supplementary Table 5) for detection with Clarity Western ECL Substrate (Bio-Rad, 1705061). Chemiluminescent signal was detected using Biorad ChemiDoc and ImageJ software.

### Immunohistological analysis

Urinary bladder tissue was embedded in paraffin and sectioned at 6 µm. For removal of soluble forms of ɑSyn from bladder tissue, paraffin-embedded slides were treated with 20 μg/mL proteinase K (PK) for 30 min after antigen retrieval. Proteinase activity was quenched with 5 mM phenylmethylsulfonyl fluoride (PMSF) for 5 min at room temperature. Sections of human brain with or without Lewy bodies were treated in parallel to ensure efficient removal of soluble ɑSyn. Staining procedure for the urinary bladder is described in supplementary methods and antibodies are listed in Supplementary table 5. Semi-quantitative analysis of pathology in human urinary bladder was performed by two observers blinded to different conditions. Scoring of pathology was in accordance with previously published protocols [11]. Brain and spinal cord tissue were frozen and collected as a series of coronal sections of 40 µm via microtome sectioning (Leica). Staining procedure for brain and spinal cord tissue is described in Supplementary methods and antibodies are listed in Supplementary table 5.

### Statistical analysis

Statistical analysis was performed using GraphPad Prism 8 software. The type of analysis with post hoc correction for multiple testing is indicated in the legend of each figure. Statistical levels were set at *p < 0.05, **p < 0.01, and ***p < 0.001.

## Supplementary Information

Below is the link to the electronic supplementary material.

Supplementary file1 (DOCX 13690 kb)

Supplementary file2 (MP4 6461 kb)

## Data Availability

All data are available in the main text or the supplementary materials.
