# Supplementary material for: Urinary tract infections trigger synucleinopathy via the innate immune response
Source: Acta Neuropathol. 2023 Mar 30;145(5):541–59. doi: 10.1007/s00401-023-02562-4 (PMC10119259; doi:10.1007/s00401-023-02562-4)
Supplement: Supplementary file 1 — Supplementary file1 (DOCX 13690 kb) [file 401_2023_2562_MOESM1_ESM.docx]

**Supplementary Figures**

**
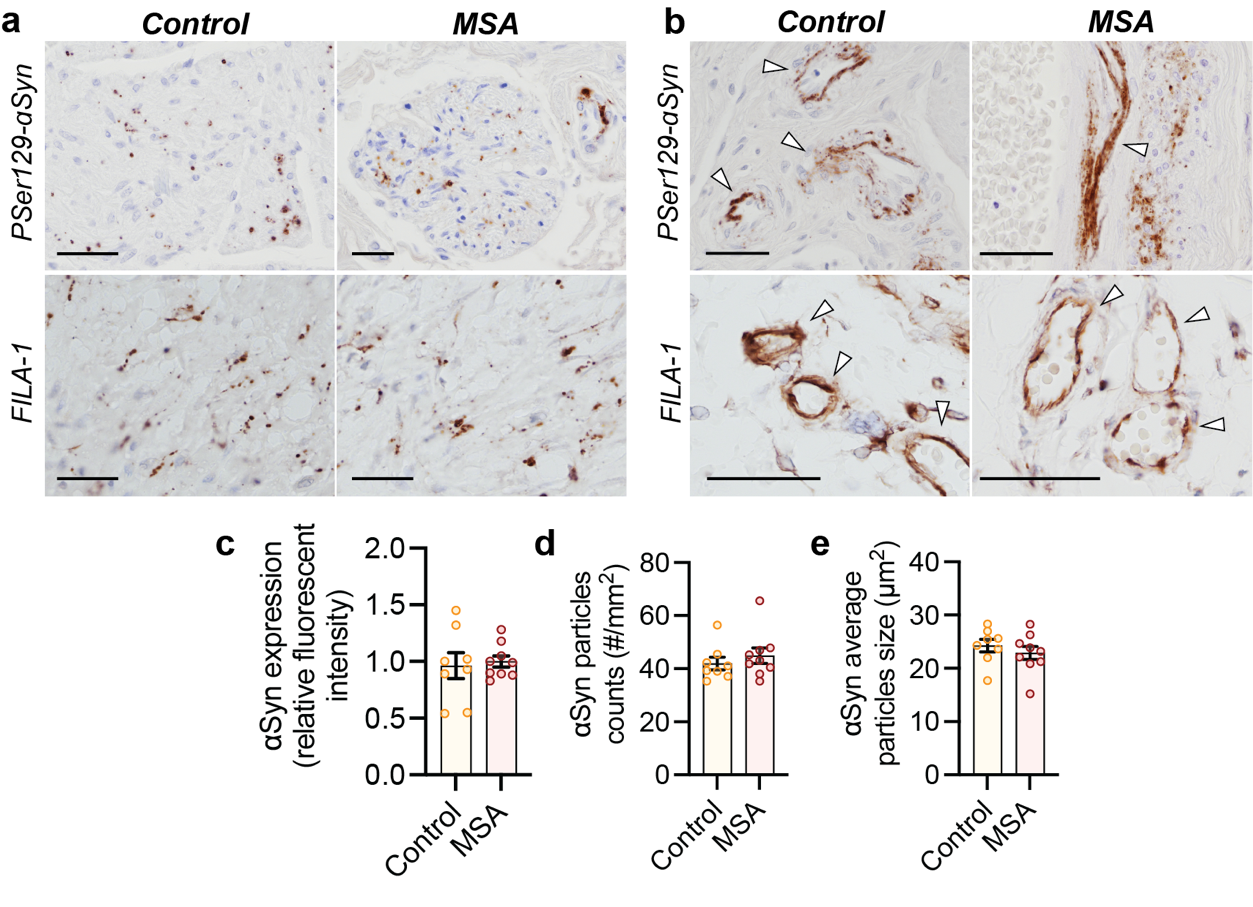
**

**Supplementary fig. 1. Detection of total ɑSyn in the urinary bladder of MSA patients and controls.** Immunohistochemical analysis of endogenous and post-translationally modified ɑSyn in paraffine-embedded human urinary bladder tissue. Heat induced epitope retrieval (HIER)-mediated antigen retrieval was performed before staining for PSer129-ɑSyn or with a conformation-specific antibody that preferentially binds aggregated forms of ɑSyn (FILA-1). (**A**) Detection of PSer129-ɑSyn (upper panel) and FILA-1 (lower panel) shows the presence of post translationally or pathological modified ɑSyn in urinary bladder of controls and MSA cases. (**B**) Pathological ɑSyn (PSer129-ɑSyn and FILA-1) is also found around blood vessels (white arrows) in urinary bladder of normal subjects and MSA cases (scale bars represent 50 µm). (**C**) Quantification of ɑSyn expression via fluorescent staining with the MJFR-1 antibody that recognizes all forms (total) ɑSyn shows equal levels of ɑSyn in control and MSA cases (n=8, p > 0.05 with unpaired two-tailed t-test) with no detectable differences between ɑSyn particle (**D**) number or (**E**) size.

**
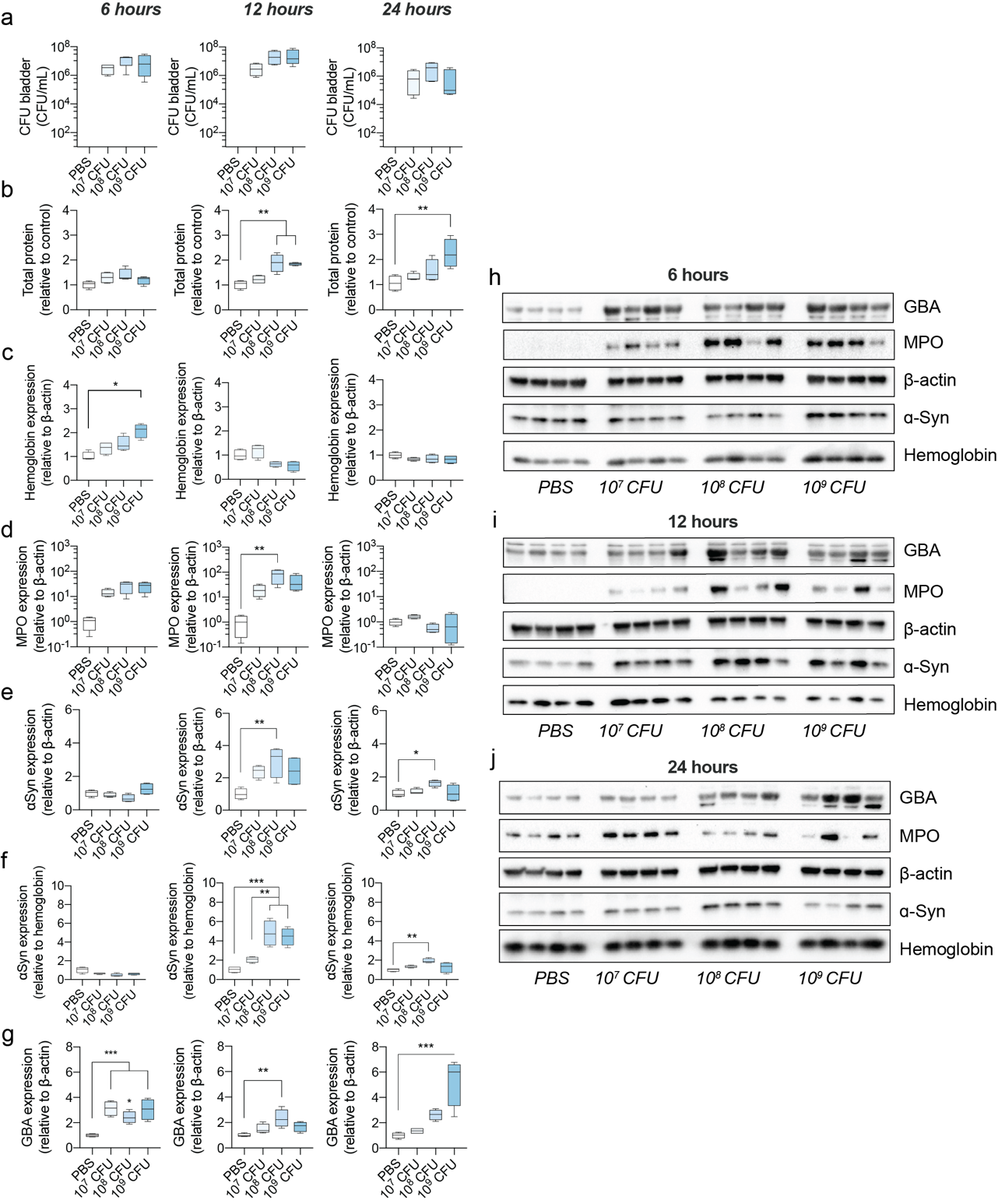
**

**Supplementary Figure 2. ɑSyn expression varies with the strength of urinary tract infection in C57BL/6N mice.** WT C57BL/6N mice were acutely infected with varying doses of UPEC and analyzed at different time points for ɑSyn expression. **a)** To determine the relative ɑSyn response to different infectious triggers, animals were infected with 10^7^, 10^8^ or 10^9^ CFUs. Bladders isolated at 6, 12 and 24 hours showed similar infectious titers in urinary bladder of infected animals. **b)** Urinary bladders showed increased total protein content towards later time points suggestive of an active immune response. **c)** Relative levels of hemoglobin (versus β-actin) increase at the 6-hour time point in a dose-dependent way but remains unaltered during the later course of infection (n = 4, p* < 0.05 with one-way ANOVA and Bonferroni correction for multiple comparisons). **d)** MPO expression during different time points indicate neutrophil infiltration at earlier time points, with a peak at 12 hours of infection for 10^8^ CFU (n = 4, p** < 0.01 with Kruskal Wallis and Dunn’s correction for multiple comparisons). **e)** Analysis of ɑSyn expression reveals increased levels at the 12- and 24-hour time points with the two higher injected doses of 10^8^ and 10^9^ CFU but not with 10^7^ CFU (n = 4, p** < 0.01 with one-way ANOVA and Bonferroni correction for multiple comparisons), **f)** which is significant after correcting for blood erythrocytes, an abundant source of ɑSyn (n = 4, p*** < 0.001 with one-way ANOVA and Bonferroni correction for multiple comparisons). This indicates that the strength of the trigger determines ɑSyn expression levels. **g)** The lysosomal enzyme GBA, and PD risk factor, enriched in peripheral blood mononuclear cells and neutrophils is significantly elevated during the course of infection (n = 4, p*** < 0.001 with one-way ANOVA and Bonferroni correction for multiple comparisons). **h-j)** Western Blot analysis of GBA indicates different glycosylated forms of the protein, with the lower bands suggesting removal of glycosylation and a shift to a lysosomal active form of the enzyme. Western blots from graphs at different time points and different doses **a-g)** are shown in **h-j)**.

**
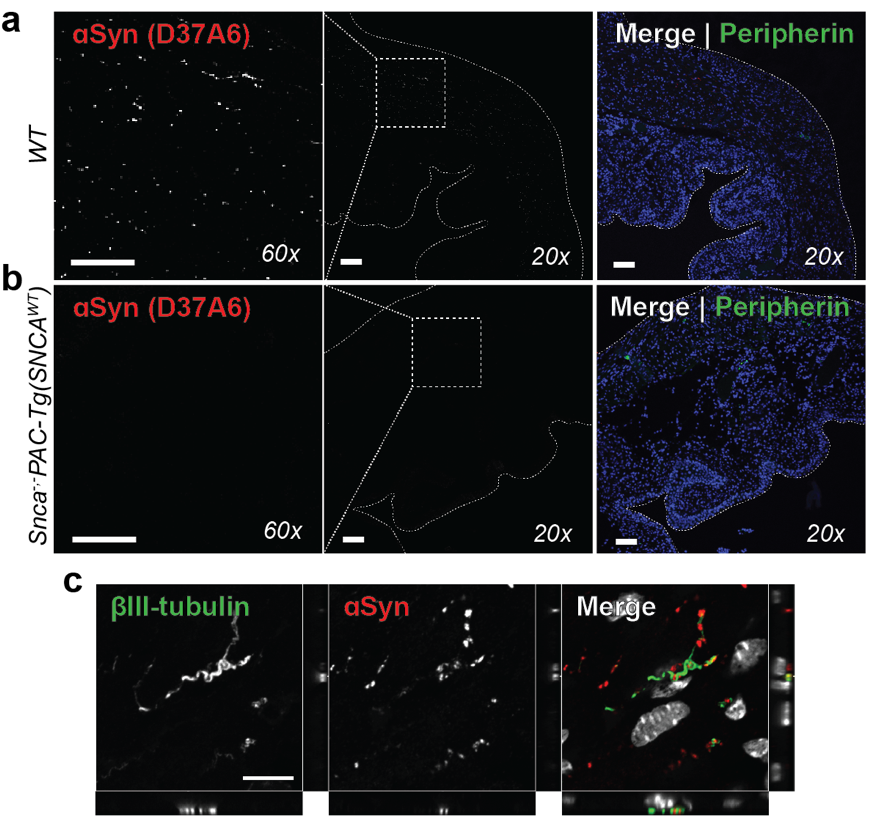
**

**Supplementary Figure 3. ɑSyn expression in urinary bladder of WT mice.** Coronal sections of urinary bladder of C57BL/6N mice were analyzed for endogenous ɑSyn expression. **a)** Left and middle panels show detection of ɑSyn using the D37A6 antibody that specifically recognizes rodent ɑSyn in urinary bladder of WT mice at 60x and 20x magnifications, respectively. Right panel shows a 20x overview of the urinary bladder with DAPI, ɑSyn and peripherin expression. Scale bar is 50 µm. **b)** Detection of ɑSyn in transgenic human ɑSyn mice is negative (hu-ɑSyn mice are knock out for rodent ɑSyn) using the D37A6 antibody, indicating that ɑSyn expression is specific for mouse ɑSyn in urinary bladder. **c)** Expression of ɑSyn was detected in βIII tubulin-positive neurons of the detrusor muscle. Scale bar is 15 µm.

**
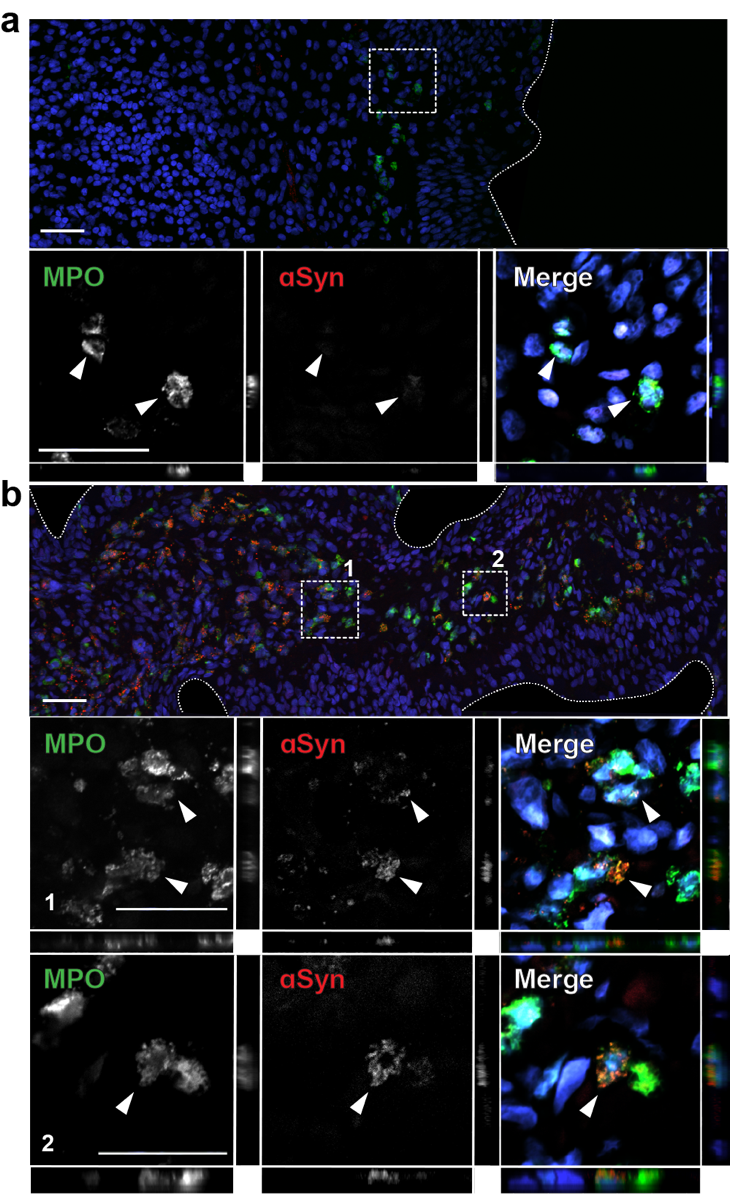
**

**Supplementary figure 4. Detection of ɑSyn in neutrophils of human urinary bladder during cystitis. a)** ɑSyn expression is low in resting neutrophils in the lamina propria of normal urinary bladder. Inset shows MPO-positive neutrophils and low levels of ɑSyn. Scale bar is 40 µm. **b)** A case of cystitis with edematous urothelium and widespread infiltration of neutrophils. Inset 1 shows ɑSyn deposited with MPO and decondensation of DAPI. Inset 2 shows an intact neutrophil with overlap between ɑSyn and MPO. Scale bar is 40 µm.

**
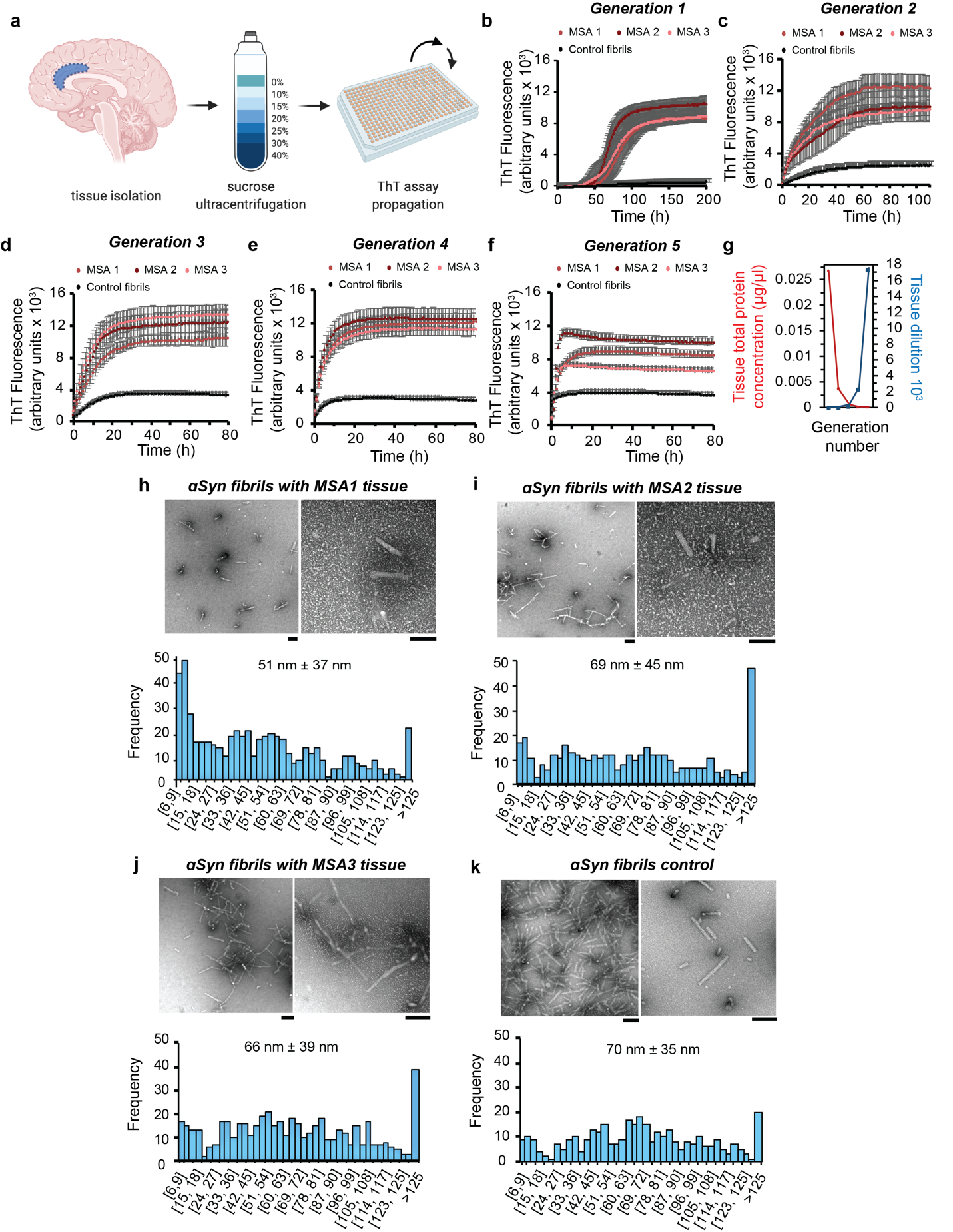
**

**Supplementary Figure 5. Amplification of fibrils from brain tissues of patients with MSA. a)** Anterior cingulate tissues of three individuals with MSA were used to generate ɑSyn fibrils for the mouse-injection studies. Homogenized brain tissue was fractionated via sucrose ultracentrifugation and amplified using 384-well plates. Four to six wells were supplemented with 10 μM ThioflavinT (ThT) to monitor the progress of fibril formation. **b**) Fibril formation of recombinant ɑSyn was seeded with 10%-sucrose-MSA fractions. Control samples are fibrils formed by incubation of ɑSyn only. Fibrils were propagated in five sequential reactions (**b-f**), and each fibril generation was formed by seeding ɑSyn polymerization with the fibrils collected from the previous reaction. MSA1, MSA2, and MSA3 - 10%-sucrose fractions of anterior cingulate tissues of three patients diagnosed with MSA (means = 4-6 technical replicates, SE). **g**) Total protein concentration of MSA–tissue seeds (left) and times dilution (right) are plotted for each generation of fibrils. Transmission electron microscopy images of **h**) MSA patient 1-, **i**) MSA patient 2-, **j**) MSA patient 3-, tissue-seeded and **k**) ɑSyn -only fibrils (mean $\pm$ standard deviation is indicated for each condition). Average length of sonicated fibrils measured in the electron micrographs are indicated for each patient. Animals were injected with samples in which three fibril batches generated with tissues of three MSA patients were combined in equimolar ratios. Scale bar is 100 nm.

**
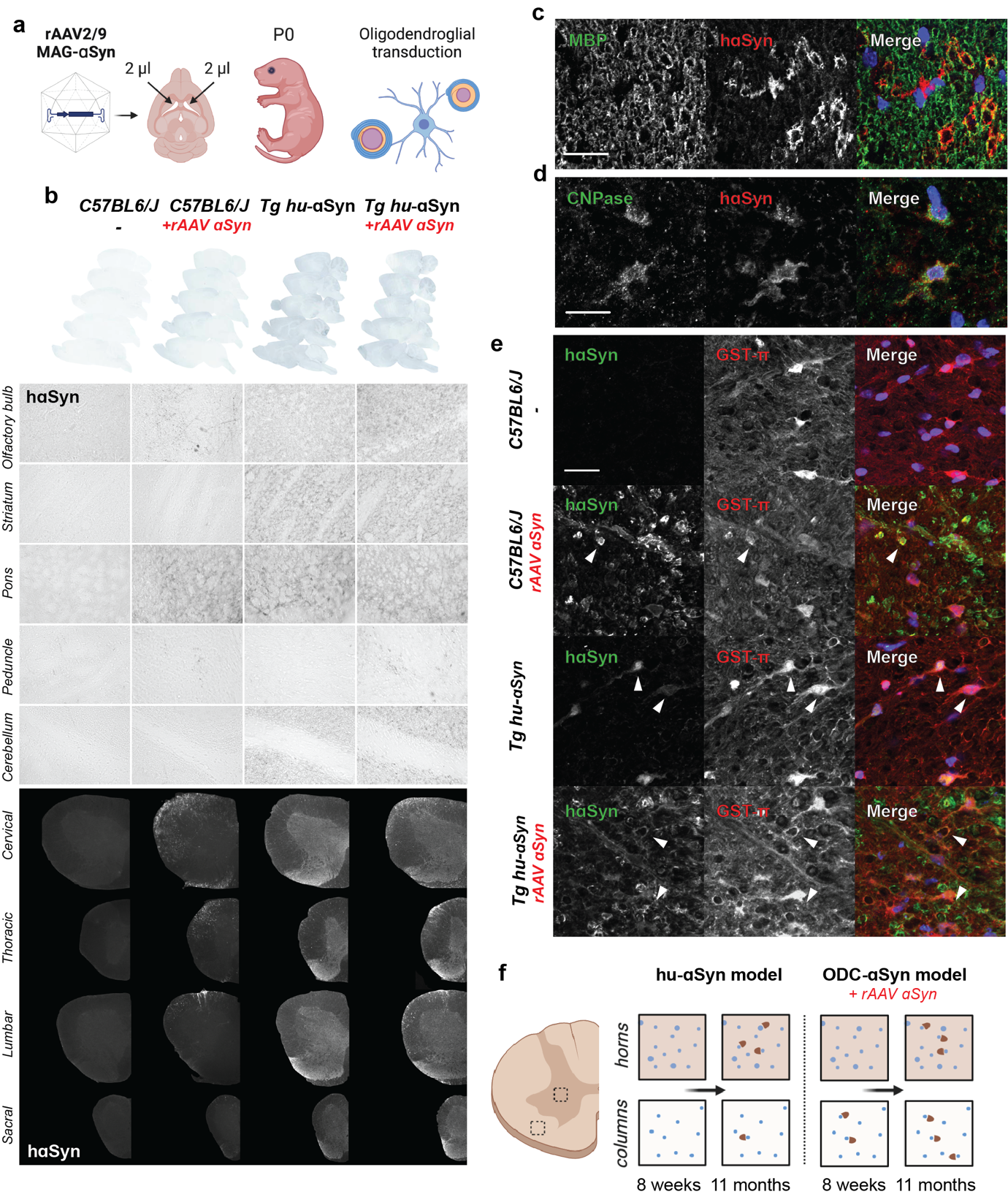
**

**Supplementary Figure 6. Animal models used to study MSA disease progression *in vivo*.** **a)** Mouse neonates (P0) were bilaterally injected in the lateral ventricles with the oligodendrocyte expressing viral vector rAAV2/9-MAG ɑSyn. The vector expresses in oligodendrocytes with a specificity of over 95%. **b)** After intracebroventricular (ICV) delivery in C57BL/6J mice, there is detection of human ɑSyn with the human ɑSyn-specific antibody MJFR-1 in bulbus olfactorius, pons neuropil and the cerebellar peduncle of the cerebrum 21 days after injection. The expression of human ɑSyn from transgenic human ɑSyn mice (Tg(SNCA^WT^) and the ICV delivered rAAV2/9 MAG-ɑSyn in mouse cerebrum cannot be distinguished. Closer examination of the spinal cord of C57BL/6J and transgenic human ɑSyn mice reveals that ICV delivery of the viral vector results in widespread ɑSyn expression in the anterior columns of the spinal cord. Confocal analysis of the mature oligodendroglial markers **c)** MBP and **d)** CNPase with and human ɑSyn shows that the expression of human ɑSyn is restricted to mature spinal cord oligodendrocytes (Scale bar indicates 40 µm). **e)** Oligodendroglial expression of human ɑSyn is absent in spinal cord of WT C57BL/6J mice but stain positive for C57BL/6J mice transduced with rAAV2/9 MAG-ɑSyn as indicated by the oligodendroglial marker GST-π (scale bar indicates 50 µm). Transgenic human ɑSyn mice express detectable levels of oligodendroglial ɑSyn under basal conditions and ICV injection with rAAV2/9 MAG-ɑSyn results in more widespread expression of human ɑSyn in spinal cord oligodendroglial cell bodies and myelin tracts of the anterior columns **f)** Summary of the two models used to study disease progression in MSA. Transgenic human ɑSyn mice (the hu-ɑSyn model) and transgenic human ɑSyn mice injected with rAAV2/9 MAG-ɑSyn (the ODC-model) differentially express oligodendroglial human ɑSyn. The hu-ɑSyn model expresses relatively low levels of oligodendroglial human ɑSyn at 8 weeks and progressively develops MSA-like features. The ODC-ɑSyn model expresses ɑSyn in anterior column at 8 weeks and progressively develops additional pathology including myelin loss and spinal GCIs.

**
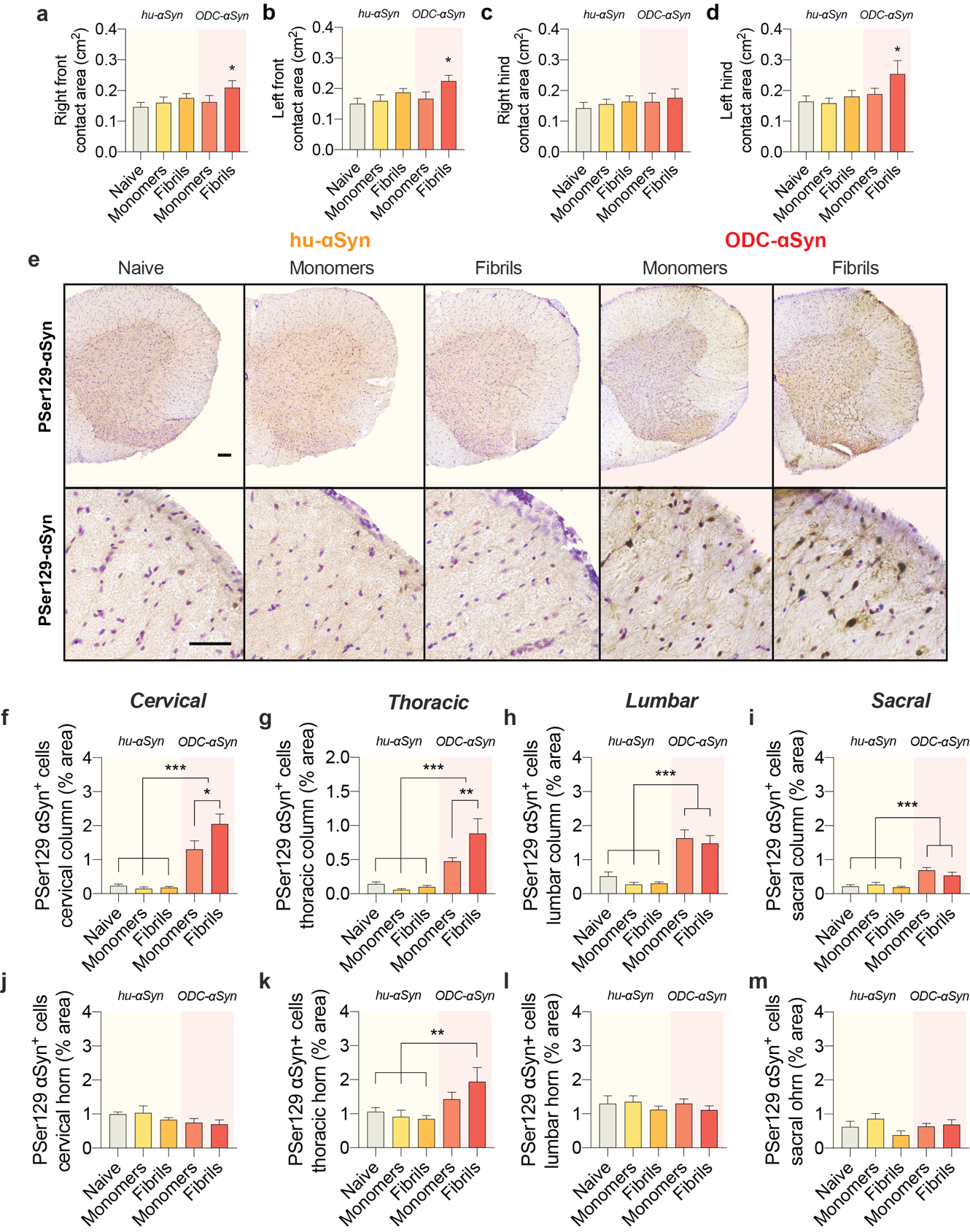
**

**Supplementary Figure 7. Characterization of behavioral deficits and pathological markers after injection of MSA fibrils in mouse urinary bladder.** Animals were injected with MSA fibrils or ɑSyn monomers at 8 weeks of age and followed up for behavioral deficits. At 9 months after injection of MSA fibrils or monomers, fine motor control deficits in **a)** right and **b)** left forepaws and **c)** right and **d)** left hindpaws are apparent in ODC-ɑSyn mice injected with MSA fibrils but not after injection with monomers (n ≥ 6, s.e.m. *p<0.05 with mixed-effects analysis of two-way ANOVA and Tukey post hoc correction for multiple comparison). No motor deficits are observed when injecting recombinant protein in hu-ɑSyn mice. **e)** Analysis of PSer129-ɑSyn inclusions in Nissl-stained mouse spinal cord (scale bar 100 µm) quantified in **f-m)** shows increased synucleinopathy in spinal cord white matter columns of the **f)** cervical and **g**) thoracic segments and the thoracic anterior horn compared to ODC-ɑSyn injected with monomers or control animals (n ≥ 6, s.e.m. *p<0.05 with mixed-effects analysis of two-way ANOVA and Tukey post hoc correction for multiple comparison). The increase in PSer129-ɑSyn immunoreactivity in ODC-ɑSyn animals compared to hu-ɑSyn mice injected with monomers could be due to viral vector mediated expression of human ɑSyn in oligodendrocytes. No additional pathology is observed in hu-ɑSyn mice injected with monomers of MSA fibrils.

**
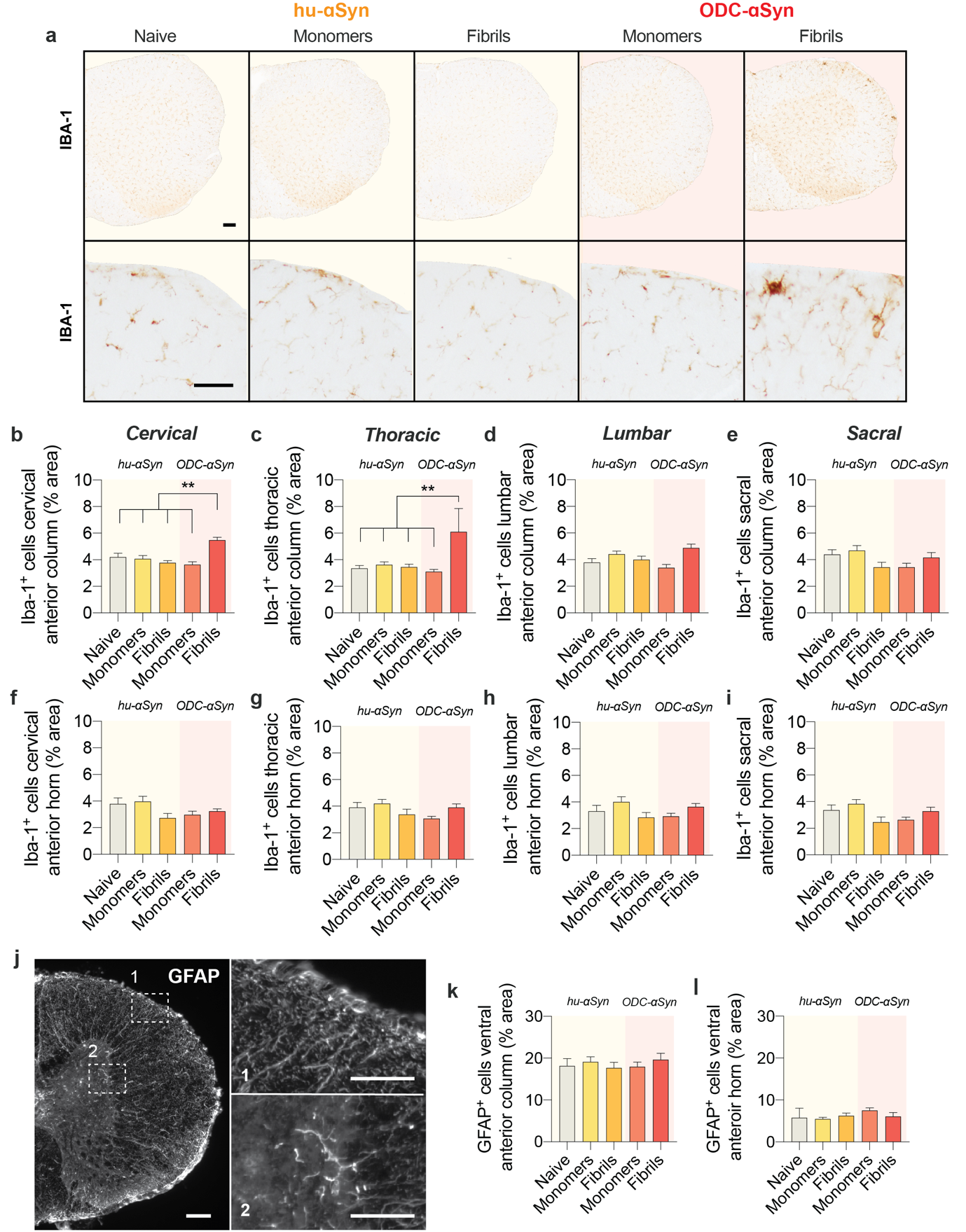
**

**Supplementary Figure 8. Characterization of inflammatory markers in spinal cord after injection of MSA fibrils in mouse urinary bladder. a)** Immunohistochemical analysis of Iba-1 in spinal cord (scale bar 100 µm) quantified in **b-i**) shows microglial activation in the spinal cord white matter anterior columns of cervival and thoracic segments of ODC-ɑSyn animals injected with MSA fibrils (n ≥ 6, s.e.m. *p<0.05 with mixed-effects analysis of two-way ANOVA and Tukey post hoc correction for multiple comparison). No microglial activation was observed ODC-ɑSyn animals injected with monomers or in any other experimental conditions. **j)** Analysis of GFAP in spinal cord (200 µm scale bar) shows no differences in astrocyte response in the **k)** thoracic white matter or **l)** thoracic anterior horn between different experimental conditions.

**
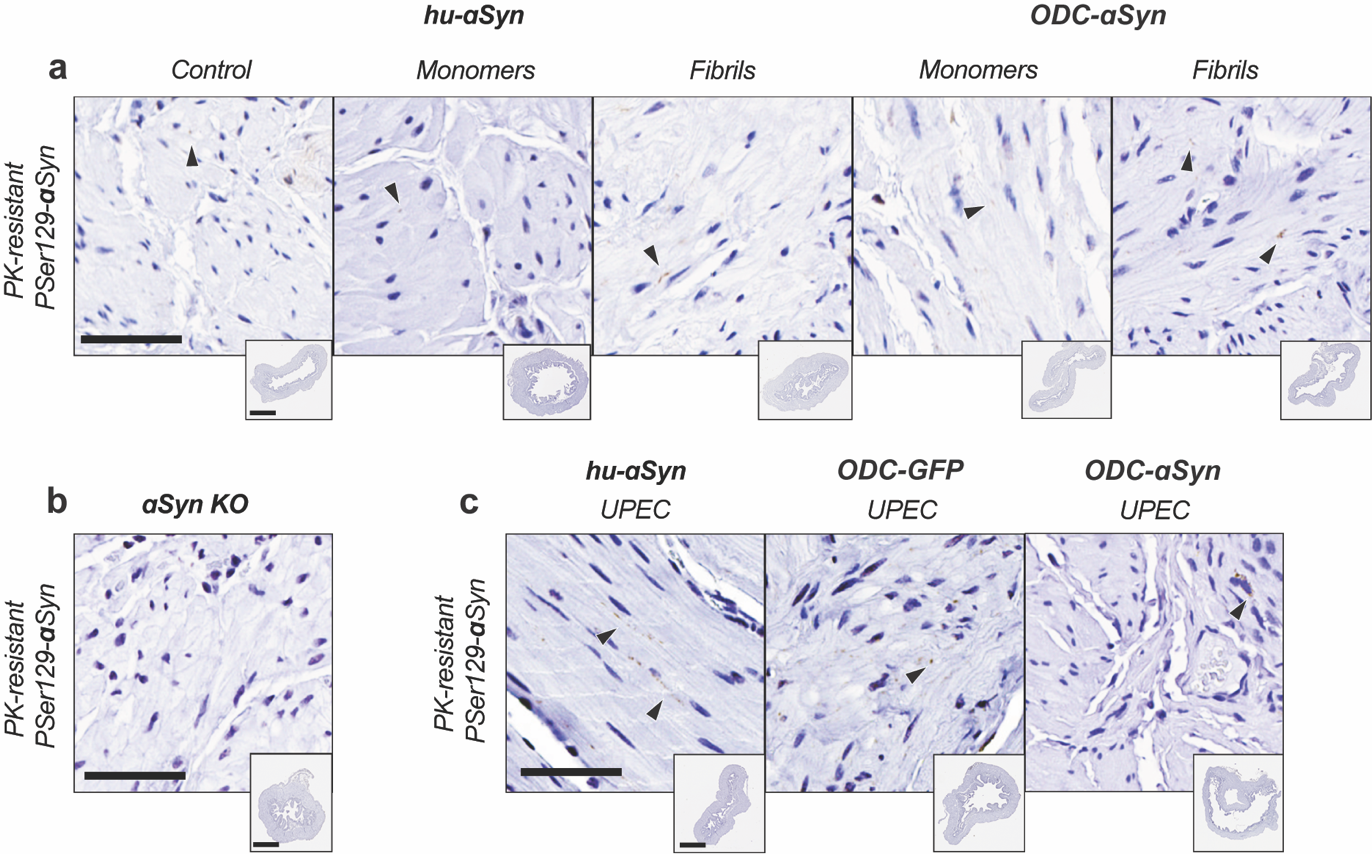
**

**Supplementary figure 9. Faint detection of aggregated PSer129-ɑSyn in urinary bladder after recombinant ɑSyn injection**. At the final experimental time point urinary bladders were paraffin-embedded and treated with PK to remove all soluble forms of ɑSyn. Urinary bladders were treated with 0.1% cresyl violet before staining. (**A**) Staining for the pathological marker PSer129-ɑSyn reveals diffuse but faint granular staining in control conditions and conditions injected with recombinant ɑSyn monomers or MSA fibrils. (**B**) No detectable granular staining was observed for KO animals. (**C**) A diffuse staining was also observed for animals infected with UPEC. No apparent differences were detected between any of the experimental conditions (scale bar is 50 µm for upper panels and 1 mm for overview panels). Black arrowsheads indicate staining of PK-resistant PSer129-ɑSyn puncta.

**Supplementary Materials**

***Epidemiological study of the impact of urinary tract infection on Multiple System Atrophy***

A population-based case-control study of urinary tract infection (UTI) before diagnosis of Multiple System Atrophy (MSA). The study population consisted of all 108 patients diagnosed with MSA and 1080 sex-, age-, and cohabitation-matched controls randomly selected from the danish population between 2016 and 2018. We measured the association between UTI and MSA using odds ratios (OR) and 95% confidence intervals (95% CI).

***Setting***

Denmark is a high-income country with approximately 5.8 million citizens. The Danish healthcare system is publicly funded by taxation and is free at the point of care to all residents with universal coverage, except for some co-payments for prescription drugs.

## Data sources

Data was retrieved from a database established at Statistics Denmark by linkage of data from four national registers on sociodemographic factors and three clinical registers for the years 2003-2018. Using a unique personal identification number (CPR-number), we were able to link the demographic data with the clinical data and information on redeemed prescriptions from community pharmacies. The CPR-number is given to each Danish citizen at birth or immigration. The sociodemographic registers include information on date of birth, vital status, sex (binary), civil status, family, citizenship, municipality, region, income, education, and housing. The Danish National Patient Register (DNPR) contains individual-level healthcare information on all inpatient contacts, contacts and emergency department contacts, surgeries and other procedures. The Register of Medicinal Product Statistics (RMPS) contains individual-level information on all prescriptions redeemed from Danish pharmacies, including the personal identification number, dispensing date, anatomic therapeutic chemical code (ATC), number of packages, number of defined daily doses, product name, and indication.

## Study population

We included all adults (18 years of age or older) registered as living in Denmark between 1 January 2016 and 31 December 2018. Multiple system atrophy (MSA) was defined as a registration in DNPR with a confirmed diagnosis with ICD-10 code G23.2 (Multiple system atrophy, parkinsonian type) and G23.3 (Multiple system atrophy, cerebellar type). For each case we randomly sampled ten controls among all (n=1.782.019) potential persons in the background population with same sex, birthdate +/- 90 days, and cohabitation status. Cohabitation status was defined as single or living together defined as two adults living at the same address and are married, in registered partnership, having children together, or with an age difference lower than 15 years.

***Definition of UTI***

Any redemption within 14 days of end of treatment of an UTI (based on redemption date and daily defined dose) is considered as one UTI. Recurrent UTI was defined as three or more UTI within a year, two or more UTI within six months.

## Ethics

Under Danish law, studies using pre-existing, routinely collected data from registers, do not require ethical approval or informed consent. The project was approved by Statistics Denmark (project number: 707838) and the Data Protection Agency (P-2019-616).

## Data availability

Because of data protection regulation, register data cannot be shared directly by the authors. Data is accessible to researchers from an authorised institution after application to the Danish Health Data Authority and Statistics Denmark.

## Flow chart of the case-control study population


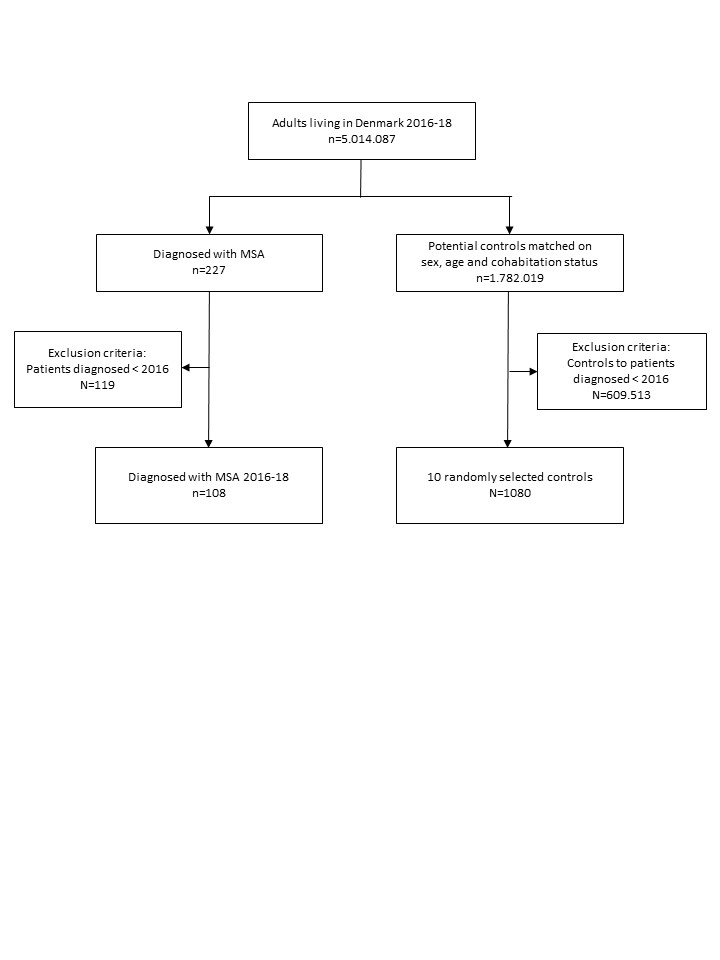


***Immunohistochemistry***

Urinary bladder tissues were stained on glass mounting slides with 1 mL of incubation solution. Slides were heated at 60˚C on a heating block for 15 min and rehydrated via serial rehydration in xylene, 100% ethanol, 90% ethanol and 70% ethanol and distilled water. Antigen retrieval for urinary bladder was performed on paraffin-embedded urinary bladder tissue with the universal HIER antigen retrieval reagent (Abcam) for 30 minutes in a steam cooker at 95-100˚C. For immunofluorescent analysis, sections were blocked with 10% donkey serum (Millipore-Sigma) in 0.1% Triton-X in PBS for 30 min at room temperature. For antigen detection, different concentrations of primary antibodies are listed in **Supplementary Table 5** and were used at 4˚C overnight. Sections were triple washed in 0.1% Triton-X in PBS and incubated with secondary antibody (**Supplementary Table 5**) and 1:1000 DAPI for two hours at room temperature after which the sections were washed again with 0.1% Triton-X in PBS. Urinary bladder tissues were treated with Trueblack Lipofuscin Autofluorescent Quencher (Biotium) for 3 minutes to quench non-specific extracellular fluorescence and cellular autofluorescence. Slides were sealed with Vectashield Antifade Mounting Medium (Vector Laboratories). Images were acquired using an A1plus-RSi Scanning Confocal microscope (Nikon). For DAB immunoprecipitation and antigen detection, samples were blocked with 10% goat serum for 1 h after which they were incubated with primary antibody listed in **Supplementary Table 5** at 4˚C overnight. Next day, slides were triple washed with 0.1% Triton-X in PBS and incubated with biotinylated anti-rabbit antibody (Vector Laboratories, BA-1000-1.5) for 2 hours at RT, triple washed again and treated with Vectastain ABC kit (Vector Laboratories, PK-4000). Antigen detection was performed with Vector DAB (Vector Laboratories, SK-4100). In case of Nissl staining, slides were treated with 0.1% cresyl violet solution for 8 minutes before dehydration. After dehydration, slides were coverslipped with Cytoseal 60 mounting medium (Thermo Fisher Scientific). The sections were viewed under a Nikon Eclipse Ni-U microscope (Nikon); images were captured with a color Retiga Exi digital CCD camera (QImaging) using NIS Elements AR 4.00.08 software (Nikon)

For immunofluorescent analysis, single brain or spinal sections or a series of free-floating tissue sections (every 240 µm) were blocked with 10% donkey or goat serum (Millipore) and 4% bovine serum albumin in 0.1% Triton-X in PBS for 2 h at room temperature. Samples were incubated with primary antibody listed in **Supplementary** **Table 5** at room temperature overnight. Next day, slides were triple washed with 0.1% Triton-X in PBS and incubated with secondary antibody (**Supplementary Table 5**) for two hours at room temperature after which the sections were washed again with 0.1% Triton-X in PBS. Slides were sealed with Vectashield Antifade Mounting Medium with DAPI (Vector Laboratories). The sections were viewed under a Nikon Eclipse Ni-U microscope (Nikon); images were captured with a color Retiga Exi digital CCD camera (QImaging) using NIS Elements AR 4.00.08 software (Nikon) or a confocal microscope (Nikon A1plus-RSi Laser Scanning Confocal Microscope). For immunohistochemistry, a series of free-floating tissue sections were stained using a primary antibody listed in **Supplementary** **Table 5** and biotinylated secondary sera at 1:500 (**Supplementary** **Table 5**). For the detection of the antibody with DAB, we used a standard peroxidase-based method (Vectastain ABC kit and DAB kit; Vector Laboratories). After dehydration, slides were coverslipped with Cytoseal 60 mounting medium (Thermo Fisher Scientific). Slides were scanned using an Aperio AT2 scanner (Leica).

**Supplementary tables**

**Supplementary Table 1**. Codes for the definition of urinary tract infection (UTI)

| Register | Classification | Code | Code description |
| --- | --- | --- | --- |
| Danish National Patient Register | ICD-10 | N39.0 | Urinary tract infection, site not specified |
|  | ICD-10 | N10 | Pyelonephritis |
|  |  | N11 | Chronic prostatitis |
|  | ICD-10 | N30 | Cystitis |
|  | ICD-10 | N34 | Urethritis |
|  | ICD-10 | O23 | Infections of genitourinary tract in pregnancy |
|  | ICD-10 | O862 | Urinary tract infection following delivery |
|  | ICD-10 | T814U | Post-operative urinary tract infection |
| Register of Medicinal Product Statistics | ATC | J01CA08 | Pivmecillinam |
|  | ATC | J01EA | Trimethoprim |
|  | ATC | J01EB | Sulphonamides |
|  | ATC | J01XE | Nitrofurantoin |
|  | Indication | 103 | Cystitis |
|  | Indication | 104 | Urinary tract infection |
|  | Indication | 126 | Chronic urinary tract infection |
|  | Indication | 265 | Prevention of urinary tract infection |
|  | Indication | 449 | Prostatitis |

ATC; Anatomic Therapeutic Chemical Code, ICD; International classification diseases

**Supplementary Table 2**. Sex stratified analysis of having at least one UTI in the specified period before MSA diagnosis/match date

| Analysis | Females | | |  | Males | | |
| --- | --- | --- | --- | --- | --- | --- | --- |
|  | **MSA-cases (%)** | **Controls**  **(%)** | **OR**  **(95% CI)** |  | **MSA-cases (%)** | **Controls (%)** | **OR**  **(95% CI)** |
| 2-8 years | 52  (48.2 %) | 253  (23.4 %) | 3.04 (2.03-4.54) |  | 23  (41.1 %) | 87  (15.5 %) | 3.79 (2.12-6.76) |
| 2-4 years | 45  (41.7 %) | 180  (16.7 %) | 3.57 (2.36-5.41) |  | 20  (35.7 %) | 61  (10.9 %) | 4.55 (2.48-8.35) |
| 5-8 years | 24  (22.2 %) | 149  (13.8 %) | 1.79 (1.10-2.90) |  | 9  (16.1 %) | 44  (7.9 %) | 2.25 (1.03-4.88) |
| Relapse  2-8 years | 26  (24.1 %) | 84  (7.8 %) | 3.76 (2.29-6.16) |  | 11  (19.6 %) | 27  (4.8 %) | 4.83 (2.25-10.36) |

**Supplementary Table 3.** Having at least one UTI in the specified period before MSA diagnosis/match date

| Analysis | MSA-cases (%) | Controls (%) | OR (95% CI) | p-value | OR (95% CI)* | p-value |
| --- | --- | --- | --- | --- | --- | --- |
| 2-8 years | 29 (55.8 %) | 166 (31.2 %) | 2.69 (1.51-4.79) | p<0.001 | 3.09 (2.06-4.63) | p<0.001 |
| 2-4 years | 25 (48.1 %) | 119 (22.9 %) | 3.12 (1.75-5.58) | p<0.001 | 3.63 (2.39-5.51) | p<0.001 |
| 5-8 years | 15 (28.9 %) | 105 (20.2 %) | 1.60 (0.85-3.03) | p=0.15 | 1.81 (1.11-2.94) | p=0.02 |
| Relapse 2-8 years | 15 (28.9 %) | 57 (11.0 %) | 3.29 (1.70-6.37) | p<0.001 | 3.91 (2.37-6.46) | p<0.001 |

* Adjusted for urosepsis in the specified period

**Supplementary table 4. Primers used for viral vector development and characterization**

| Primer | Sequence |
| --- | --- |
| MAG sense | 5’-TATATGCTAGCCCTCAGAAGGAACCAACACTGCCAGCACTT-3’ |
| MAG antisense | 5’-ATTTATGATATCGCCCCCACTTGCCAGCCCCTCCCCTCCC-3’ |
| polyA sense | 5′-TCTAGTTGCCAGCCATCTGTTGT-3′ |
| polyA antisense | 5′-TGGGAGTGGCACCTTC CA-3′ |
| polyA probe | 5′-TCCCCCGTGCCTTCCTTGACC-3 |

**Supplementary table 5. Summary of human brain tissues used to seed fibril formation of ɑSyn.**

| Diagnose | Gender | Age [years] | Brain Region | PMI* [hrs] |
| --- | --- | --- | --- | --- |
| MSA | M | 64 | Anterior cingulate | 68 |
| MSA | M | 71 | Anterior cingulate | 20 |
| MSA | F | 64 | Anterior cingulate | 4 |

*PMI - postmortem interval.

**Supplementary Table 6. Antibodies used for different applications throughout this study.**

| Antibody | Concentration | Application | Comments | Supplier | Catalog nr. (clone) |
| --- | --- | --- | --- | --- | --- |
| *Primary antibodies* | | | | | |
| Rabbit anti-ɑ-synuclein | 1:600 | IF | Detects rodent ɑSyn | Cell Signaling | 4179S (D37A6) |
| Rabbit anti-ɑ-synuclein | 1:1000 | IF | Detects human ɑSyn | Abcam | Ab138501 (MJFR-1) |
| Rabbit anti-ɑ-synuclein | 1:1000 | IHC | Preferentially binds HMW ɑSyn | Abcam | Ab209538 (MJFR14-1-1) |
| Mouse anti-ɑ-synuclein | 1:2000 | WB | Detects rodent and human ɑSyn | Abcam | 610787 (Clone 42) |
| Mouse anti- ɑ-synuclein | 1:1000 | IF | Detects human ɑSyn | ThermoFisher | 32-8100 (syn211) |
| Rabbit anti-PSer-129 ɑ-synuclein | 1:1000 | IHC/IF | - | Abcam | Ab51253 (EP1536Y) |
| Mouse anti-βIII-tubulin | 1:2000 | IF | - | BioLegend | 801201 (TUBB3) |
| Chicken anti-synaptophysin | 1:300 | IF | - | SySy | 101 006 |
| Goat anti-MPO | 1:300 | IF | - | Novus | AF3667 |
| Rabbit anti-MPO | 1:500 | WB | - | Abcam | Ab208670 (EPR20257) |
| Rabbit anti-GST-π | 1:200 | IF | - | MBL | 312 |
| Rat anti-MBP | 1:500 | IF | - | Abcam | Ab7349 (12) |
| Mouse anti-CNPase | 1:500 | IF | - | Abcam | Ab6319 (11-5B) |
| Chicken anti-GFAP | 1:2000 | IF | - | Abcam | Ab4674 |
| Rabbit anti-β actin | 1:4000 | WB | - | Abcam | Ab8227 |
| Rabbit anti-GBA | 1:500 | WB | - | Novus | NBP1-32271 |
| Rabbit anti-hemoglobin | 1:2000 | WB | - | ThermoFisher | PA5-97559 |
| Chicken anti-Perherin | 1:500 | IF | - | Aves | Per |
| Goat anti-Iba-1 | 1:1000 | IHC | - | Abcam | Ab107159 |
| *Secondary antibodies* | | | | | |
| Donkey anti-chicken FITC | 1:1000 | IF | - | ThermoFisher | SA1-72000 |
| Donkey anti-goat Alexa-488 | 1:1000 | IF | - | ThermoFisher | A-11055 |
| Donkey anti-rabbit Alexa-488 | 1:1000 | IF | - | ThermoFisher | A-21206 |
| Donkey anti-mouse Alexa-488 | 1:1000 | IF | - | ThermoFisher | A-21202 |
| Donkey anti-rat Alexa-488 | 1:1000 | IF | Cross affinity purified against mouse | ThermoFisher | A-21208 |
| Donkey anti-rabbit Alexa-555 | 1:1000 | IF | - | ThermoFisher | A-31572 |
| Donkey anti-goat Alexa-647 | 1:1000 | IF | - | ThermoFisher | A-21447 |
| Donkey anti-rabbit Alexa-647 | 1:1000 | IF | - | ThermoFisher | A-31573 |
| Donkey anti-mouse Alexa-647 | 1:1000 | IF | - | ThermoFisher | A-31571 |
| Goat anti-rabbit-biotin | 1:500 | IHC | - | Vector Laboratories | BA-1000-1.5 |
| Goat anti-rabbit-HRP | 1:1000 | WB | - | Cell Signaling | 7074S |
| Goat anti-mouse-HRP | 1:1000 | WB | - | Cell Signaling | 7076S |

IHC; immunohistochemistry, IF; immunofluorescence; WB; Western Blot
